# What is the fundamental ion-specific series for anions and cations? Ion specificity in standard partial molar volumes of electrolytes and electrostriction in water and non-aqueous solvents†
†Electronic supplementary information (ESI) available. See DOI: 10.1039/c7sc02691a



**DOI:** 10.1039/c7sc02691a

**Published:** 2017-08-21

**Authors:** Virginia Mazzini, Vincent S. J. Craig

**Affiliations:** a Department of Applied Mathematics , Research School of Physics and Engineering , The Australian National University , Canberra , ACT 2601 , Australia . Email: vince.craig@anu.edu.au

## Abstract

The importance of electrolyte solutions cannot be overstated. Beyond the ionic strength of electrolyte solutions the specific nature of the ions present is vital in controlling a host of properties. Therefore ion specificity is fundamentally important in physical chemistry, engineering and biology. The observation that the strengths of the effect of ions often follows well established series suggests that a single predictive and quantitative description of specific-ion effects covering a wide range of systems is possible. Such a theory would revolutionise applications of physical chemistry from polymer precipitation to drug design. Current approaches to understanding specific-ion effects involve consideration of the ions themselves, the solvent and relevant interfaces and the interactions between them. Here we investigate the specific-ion effects trends of standard partial molar volumes and electrostrictive volumes of electrolytes in water and eleven non-aqueous solvents. We choose these measures as they relate to bulk properties at infinite dilution, therefore they are the simplest electrolyte systems. This is done to test the hypothesis that the ions alone exhibit a specific-ion effect series that is independent of the solvent and unrelated to surface properties. The specific-ion effects trends of standard partial molar volumes and normalised electrostrictive volumes examined in this work show a fundamental ion-specific series that is reproduced across the solvents, which is the Hofmeister series for anions and the reverse lyotropic series for cations, supporting the hypothesis. This outcome is important in demonstrating that ion specificity is observed at infinite dilution and demonstrates that the complexity observed in the manifestation of specific-ion effects in a very wide range of systems is due to perturbations of solvent, surfaces and concentration on the underlying fundamental series. This knowledge will guide a general understanding of specific-ion effects and assist in the development of a quantitative predictive theory of ion specificity.

## Abstract 2

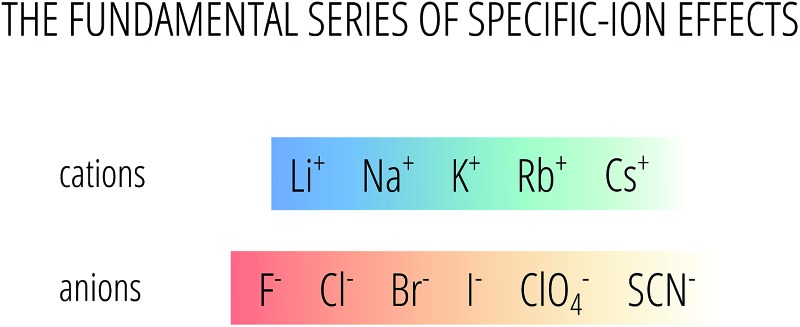
Consistent trends in ion-specificity across many solvents.

## 1 Introduction

Electrolyte solutions are ubiquitous and essential to life. Throughout science and technology electrolytes are used to control ionic strength, but beyond this the specific nature of the electrolytes is of fundamental importance. In the human body, the specific nature of a monovalent cation can mark the difference between life (and re-hydration) when NaCl is used and death (by cardiac arrest) when KCl is used.^1^ Important specific-ion effects are also observed in a vast variety of other settings, such as the processing of colloids, where the stability^2^ and rheological behaviour of the system can be controlled.^3,4^ Bubble coalescence shows rich ion specificity,^5^ as does polymer^6^ and protein stability.^7,8^ Indeed, the outcome of most measurements and experiments involving electrolyte solutions is dependent on the particular ions present.^9,10^ The effect is most often observed as a modulation in the magnitude of a property or differences in the concentration of electrolyte required to induce a change. However and notably, in some cases, different ions of the same charge induce the opposite effect. Often the strength of the effect varies regularly across the anions and cations, even in systems that are dissimilar.

The first to study specific-ion effects was Franz Hofmeister at the end of the 19^th^ century^11^ (translated from German by Kunz *et al.* ^12^). Hofmeister observed that different electrolytes had different effects on the stability of egg-white protein solutions in water: some electrolytes would destabilise them and cause the proteins to precipitate, others would instead enhance the stability of the solution. The ordering of the salts that emerged has been referred to as the “Hofmeister series”, and the phenomenon as “Hofmeister effects” or “specific-ion effects” in the following hundred years of research publications. Also the term “lyotropic series” has been used,^13–17^ although the phrase was originally introduced by Voet^18^ only for the behaviour of hydrophilic colloids in the presence of salts.

**Fig. 1:**
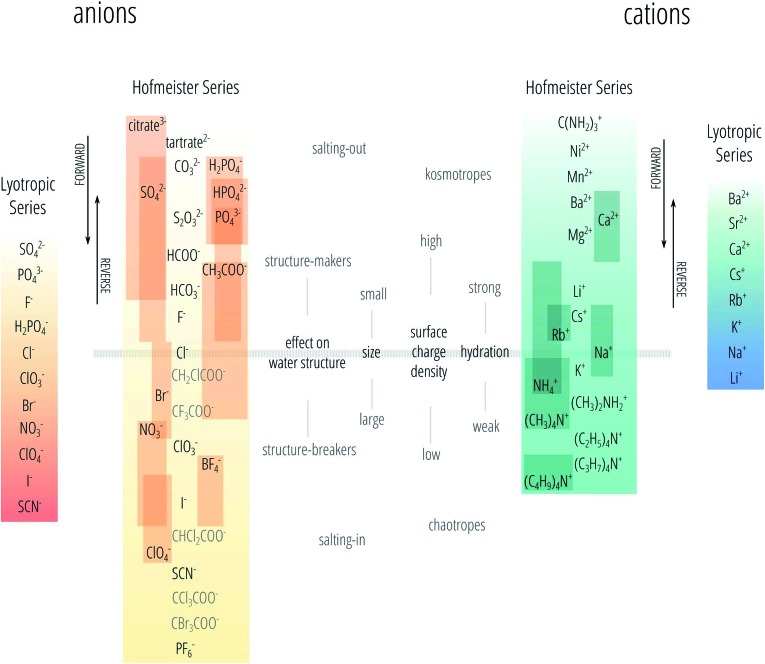
The Hofmeister and lyotropic series of ions in water. The ions of the Hofmeister series are ordered from the most (top) to least (bottom) effective in precipitating proteins out of solution. The Hofmeister series for anions was obtained by combining the series reported in ref. 9, 12 and 24–33. The Hofmeister series for cations was obtained by combining the series reported in ref. 25, 29–31 and 34–36. The ion is positioned in its most agreed upon ranking, and bars show the reported variations in position among different publications. The relative positioning of the haloacetates is well-known, but their positioning with respect to the “classic” anions of the series is certain only in a few cases. This uncertainty is reflected by presenting these ions in grey text rather than black. Ions at one end of the series are often attributed with having the opposite effect to ions at the other end of the series. As such there is a point in the series where the influence of ions reverses. The grey horizontal line traces the divide that corresponds to this property reversal. The ions of the lyotropic series, as defined by Voet,^18^ are ordered from smallest to largest lyotropic number.

The series observed and reported are not always the same as the one found by Hofmeister for the precipitation of egg albumins, but often vary and even invert depending on the experiment and experimental conditions. That is the series can reverse due to changes in pH, concentration, temperature and surface charge.^2,19–22^ Therefore, nowadays the Hofmeister series is not precisely defined and is sometimes even used when the series is not followed. Here we follow the nomenclature we proposed recently.^23^ We use the phrase “specific-ion effects” to describe all circumstances in which the type of ion has a pronounced influence on a measurable property of a solution and we reserve the definition of “Hofmeister effects” to describe the subset of specific-ion effects in which the strength of the effects of the ionic species follows the Hofmeister series. We also acknowledge the existence of the lyotropic series^18^ as a different series to the Hofmeister series, noting that sometimes these terms are used interchangeably. The Hofmeister and lyotropic series are depicted in Fig. 1. This figure attempts to account for the variability seen in reports of the Hofmeister series in the literature. The ordering of ions that is highly consistent across different studies forms the backbone of the series and is shown in the centre. The positions of other ions are indicated by a bar to show the range of positions in the series that have been reported for that ion. The various distinctions and adjectives that have been used in the literature to group the ions according to their behaviour (such as “kosmotropes/chaotropes”) are reported in the figure as well. The divide between these major groupings is usually set at chloride for anions and at sodium for cations, this is depicted by the horizontal bar.

Progress is still being made on theoretical approaches for explaining the true origins of specific-ion effects,^37–41^ though unanimity is still to be achieved.^28,42–46^ The variations observed in the ordering of specific-ion effects between different experiments and even the same experiment under different conditions hinders theorists' attempts at achieving a physical description and a mathematical formulation for specific-ion effects. It is often tacitly attributed to the influence of ions on the solvent (usually water^24,47^) and/or surfaces. Here we test the hypothesis that the ordering of specific-ion effects is fundamentally due to properties of the ions themselves and that this is exhibited experimentally under appropriate circumstances. This infers that the variations in the order of the series and the reversal of the series observed are perturbations to the underlying series due to the nature of the experiment, such as the solvent, the influence of surface properties of colloids, polymers and proteins, the concentration and pH and by the details of the measurement technique.

In order to establish the existence of a fundamental ion-specific series we expect that the series should be evident in a range of solvents. Ionic effects in non-aqueous solvents have been studied previously.^48,49^ The approach we have taken is to examine measurements where no surfaces are involved and the effect of concentration is eliminated and evaluate these measurements across as many solvents as possible. The standard partial molar volumes of electrolytes in solution and the related electrostriction are two physical quantities that satisfy these constraints: they are bulk properties of a solution; extensive and reviewed data are available across a wide range of solvents,^50,51^ and the data have been extrapolated to the limit of infinite dilution where only ion–solvent interactions apply (ion–ion interactions can be discounted).

## 2 Methods

In this work, we consider electrolytes containing the following monovalent anions and cations, that are here listed according to the Hofmeister series:AcO^–^ > F^–^ > Cl^–^ > Br^–^ > I^–^ > ClO_4_^–^ > SCN^–^Li^+^ > Cs^+^ > Rb^+^ > Na^+^ > K^+^where AcO^–^ stands for the acetate anion, CH_3_COO^–^. It must be noted that for this set of anions, the difference between Hofmeister and lyotropic series is only reflected in the position of I^–^ and ClO_4_^–^. Conversely, the ordering of the cations is very different in the two series.

## 3 Partial molar volume and electrostriction

The dependence of the standard partial molar volume on solvent properties has been investigated by Hamann and Lim^52^ in water and three other solvents: there exists an inverse linear relationship between the standard partial volume of the electrolytes and the compressibility of the solvent. Later, Marcus *et al.* ^53^ applied a stepwise multivariable linear least-squares regression to the standard partial molar volumes of ions in solution in order to relate them to solvent and ion properties. They found that the cube of the ion radius is the major contributor to the standard partial molar volume in solution. Secondly, the electron pair-sharing capability of the ion plays a role. The solvent compressibility and self-association are found to play a role but no correlation is observed with solvent dipole moment and relative permittivity. Marcus has also investigated the ion specificity of electrostriction:^54^ he analysed the correlation between the standard molar ionic electrostriction and the surface tension increment generated by ions. He found a reasonable correlation for cations but not for anions. The connection between specific-ion effects and partial standard molar volumes of ions in solution can be made in two ways. Jenkins and Marcus^55^ demonstrate that a relationship between the viscosity *B*-coefficients of the Jones–Dole equation and the molar volume of the ion in solution exists. On the other hand, the ion specificity of the viscosity *B*-coefficients has been known for a long time, starting with the work of Cox and Wolfenden.^56^ Additionally Collins^57^ accommodated it intrinsically in the formulation of his “law of matching water affinities”. This suggests that the link between electrostriction, which derives from the standard molar volumes of electrolytes, and specific-ion effects is straightforward. If such a connection can be elaborated in non-aqueous solvents, additional insight into the nature of specific-ion effects will be gained, as partial molar volumes are useful for the understanding of the interactions occurring in aqueous and non-aqueous solutions.^50^ The standard molar quantities are therefore perfectly suited for the purpose of studying specific-ion effects in non-aqueous solvents.

The partial molar volume of a species “*i*” in solution represents the change in volume of the solution upon addition of one mole of solute at constant temperature, pressure, and other components of the solution:
1

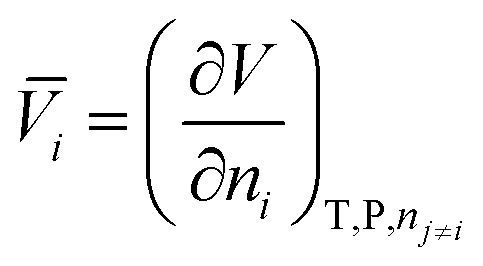

where *V*_*i*_ is the partial molar volume of the solute in solution; *V* the total volume of the solution and *n*_*i*_ the number of moles of solute present in solution. The partial molar electrostrictive volume of the species “*i*” in solution (*V*_*i* el_), can be calculated in the following way:^58^

2 *V*_*i* el_ = *V*_*i*_ – *V*_*i* intr_

where *V*_*i*_ is the experimentally derived partial molar volume of the species “*i*” in solution and *V*_*i* intr_ is the intrinsic molar volume of the species “*i*” which is not directly experimentally measurable. If the standard partial molar volume *V* <svg xmlns="http://www.w3.org/2000/svg" version="1.0" width="16.000000pt" height="16.000000pt" viewBox="0 0 16.000000 16.000000" preserveAspectRatio="xMidYMid meet"><metadata> Created by potrace 1.16, written by Peter Selinger 2001-2019 </metadata><g transform="translate(1.000000,15.000000) scale(0.005147,-0.005147)" fill="currentColor" stroke="none"><path d="M960 2600 l0 -120 -120 0 -120 0 0 -80 0 -80 -80 0 -80 0 0 -120 0 -120 -200 0 -200 0 0 -80 0 -80 200 0 200 0 0 -120 0 -120 80 0 80 0 0 -120 0 -120 120 0 120 0 0 -80 0 -80 320 0 320 0 0 80 0 80 80 0 80 0 0 120 0 120 120 0 120 0 0 120 0 120 200 0 200 0 0 80 0 80 -200 0 -200 0 0 120 0 120 -120 0 -120 0 0 80 0 80 -80 0 -80 0 0 120 0 120 -320 0 -320 0 0 -120z m640 -200 l0 -80 80 0 80 0 0 -120 0 -120 -520 0 -520 0 0 120 0 120 120 0 120 0 0 80 0 80 320 0 320 0 0 -80z m160 -600 l0 -120 -80 0 -80 0 0 -120 0 -120 -320 0 -320 0 0 120 0 120 -120 0 -120 0 0 120 0 120 520 0 520 0 0 -120z"/></g></svg> *i* is used in the calculation, we obtain the electrostrictive volume at infinite dilution, *V*∞*i* el (also known as *V*  *i* el, standard partial molar electrostrictive volume) which reflects only the interactions between the solute and the solvent (and not solute–solute effects). These quantities are the subject of the present work.

**Fig. 2:**
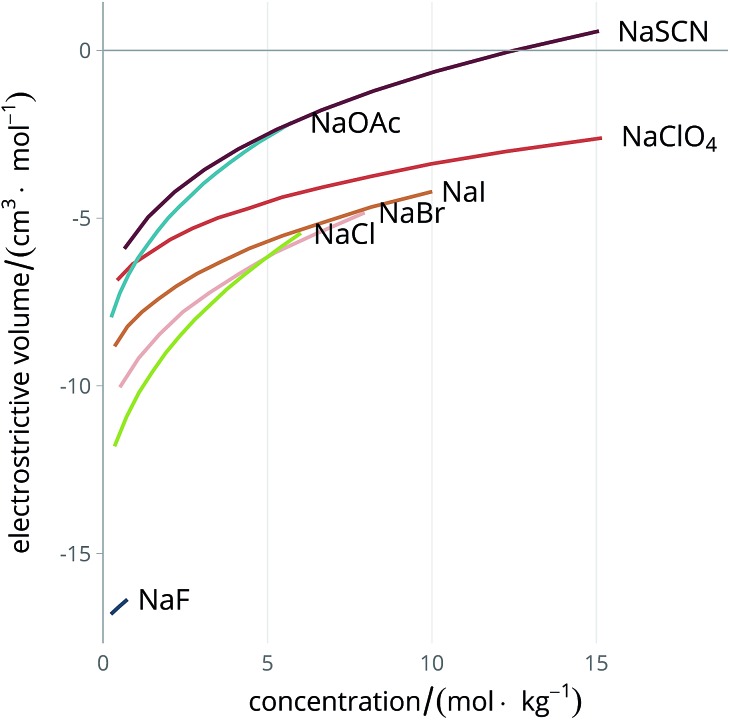
Concentration dependence of the electrostrictive volume in water for a range of electrolytes. The density of the electrolyte solutions used for the calculation of the electrostrictive volume are taken from Söhnel and Novotný.^63^

Much careful work has been done in the experimental determination of partial molal volumes of electrolytes in solution, therefore abundant information is already available in the literature, and has been extensively reviewed.^51,59^ Attempts to calculate electrostriction theoretically have also been made.^60–62^ The electrolyte exerts a constricting pressure on the order of hundreds of MPa on the surrounding solvent molecules,^58^ thus reducing the volume the solvent occupies. Fig. 2 shows the dependence of the electrostrictive volume on molal concentration in water (calculated using the density data of electrolyte solutions from Söhnel and Novotný^63^): its magnitude is different for each electrolyte. The electrostriction is larger at small concentrations as the effect saturates when all of the solvent is electrostricted therefore the solvent volume cannot be further reduced.

**Fig. 3:**
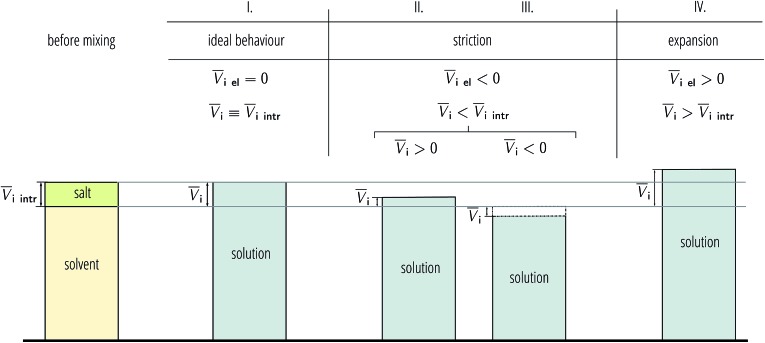
Schematic of the range of possible outcomes for the final solution volume when a salt is dissolved in a solvent depending on the effect of electrostriction. In this picture, the depicted volume of salt corresponds to one mole (therefore it represents the intrinsic molar volume). This picture particularly helps in visualising the case where the molar volume of the salt in solution is negative.

As electrostriction happens when a salt is dissolved in a solvent, the volume of the resulting solution is usually different from the sum of the volumes of the individual components, and generally smaller. This results in the partial molar volume of the electrolyte being different from the “intrinsic” volume of the electrolyte by a quantity which is the electrostrictive volume. This phenomenon is attributed to the fact that, under the huge electric field exerted by an ion, the solvent contracts (or more rarely expands). A scheme showing the dependence of the electrostrictive volume upon the reciprocal magnitude of the standard molar volume and the intrinsic molar volume is shown in Fig. 3. This phenomenon has always been connected to the electrostatic field density of the ion. Detailed treatment of partial molar volumes and electrostriction has been performed in previous reviews.^50,51^

We calculate the molar electrostrictive volume according to eqn (2), using the standard partial molar volume in order to obtain the standard partial molar electrostrictive volume (hence in the limit of infinite dilution):

3 *V*

*i* el = *V*

*i* – *V*_*i* intr_

where *V*  *i* is the standard partial molar volume of the species “*i*” in solution, and *V*_*i* intr_ is the intrinsic molar volume of the species “*i*”. The criteria we used for selecting the values of standard and intrinsic molar volumes of electrolytes are detailed in the subsection “Data sources and selection criteria”.

**Fig. 4:**
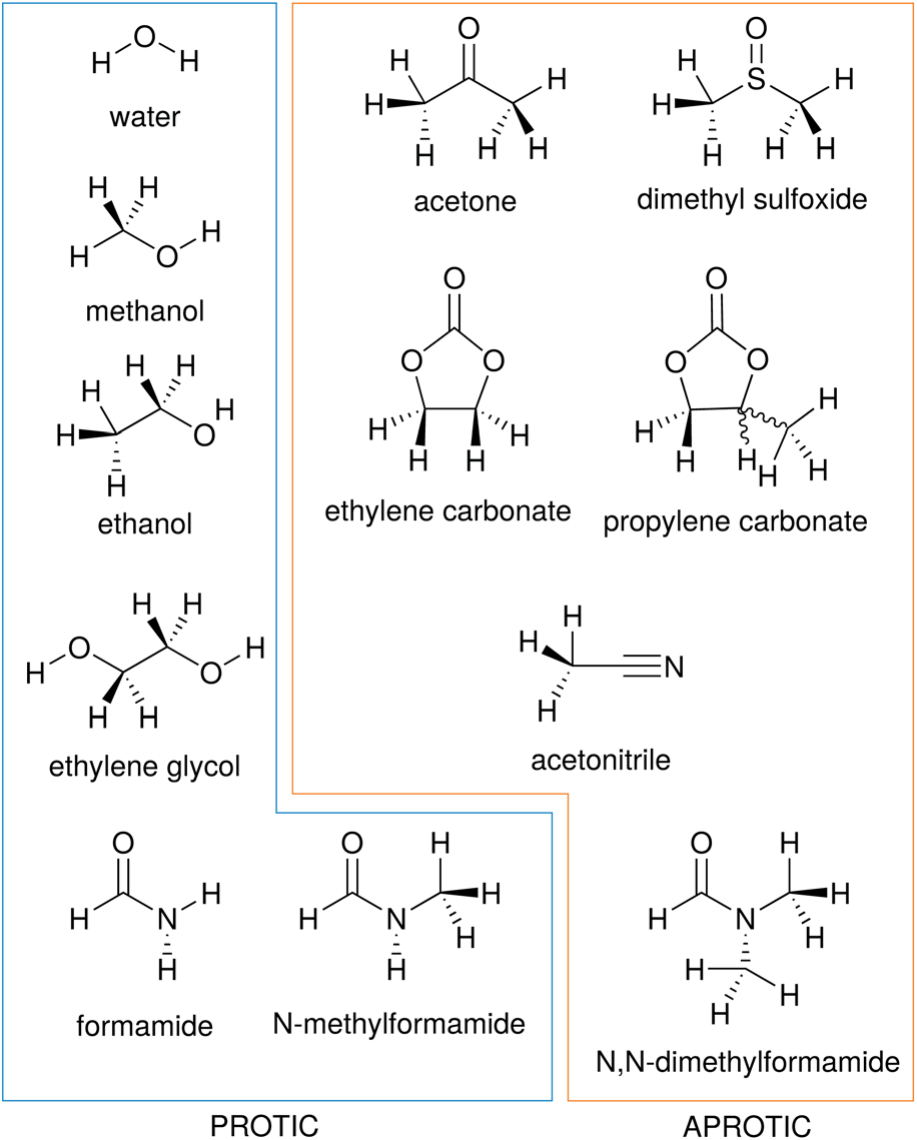
Structural formulas of the solvents used in this study.

In this work we consider the electrostrictive volume of electrolytes in water and 11 non-aqueous solvents, five of which are protic (methanol, MeOH; ethanol, EtOH; formamide, FA; *N*-methylformamide, NMF, and ethylene glycol, EG) and the remaining aprotic (dimethyl sulfoxide, DMSO; propylene carbonate, PC; ethylene carbonate, EC; acetone, ACE; acetonitrile, MeCN; *N*,*N*-dimethylformamide, DMF). Their structural formulas are reported in Fig. 4. These non-aqueous solvents cover a wide range of relative permittivities *ε*_R_ and compressibilities *κ*_T_, as shown in Table 1.

**Table 1:** Physical properties of the solvents used in this study^*a*^

Solvent	Abbreviation	*M*/(g mol^–1^)	*ρ*/(g ml^–1^)	*ε*_R_	*μ*/D	*η*/mPa s	*κ*_T_/GPa^–1^	Hydrogen bonding
Water	Water	18.015	0.9970^25^	80.100	1.8546	0.890	0.459	Protic
Methanol	MeOH	32.042	0.7914^20^	33.0	1.7	0.544	1.214	Protic
Ethanol	EtOH	46.068	0.7893^20^	25.3	1.69	1.074	1.119	Protic
Ethylene glycol	EG	62.068	1.1135^20^	41.4	2.36	16.06	0.364	Protic
Formamide	FA	45.041	1.1334^20^	111.0	3.73	3.34	0.399	Protic
*N*-Methylformamide	NMF	59.067	1.011^19^	189.0	3.83	1.678	0.577^*b*^	Protic
*N*,*N*-Dimethylformamide	DMF	73.094	0.9445^25^	38.25	3.82	0.794	0.627^*b*^	Aprotic
Acetonitrile	MeCN	41.052	0.7857^20^	36.64	3.925	0.369	1.10^*c*^	Aprotic
Acetone	ACE	58.079	0.7845^25^	21.01	2.88	0.306	1.262	Aprotic
Dimethyl sulfoxide	DMSO	78.133	1.1010^25^	47.24	3.96	1.987	0.523^*d*^	Aprotic
Ethylene carbonate	EC	88.062	1.3214^39^	89.78	4.81^*e*^	1.925^40^ ^*f*^	0.435^*g*^	Aprotic
Propylene carbonate	PC	102.089	1.2047^20^	66.14	5.36^*e*^	2.5120^*h*^	0.590	Aprotic

^*a*^ *M*: molar mass; *ρ*: density at the temperature in °C indicated by the superscript; *ε*_R_: dielectric constant; *μ*: dipole moment; *η*: viscosity at 25 °C, unless otherwise indicated by the superscript; *κ*_T_: isothermal compressibility at 20 °C. Data from the CRC Handbook of Chemistry and Physics,^64^ except for:

^*b*^Easteal and Woolf;^65^

^*c*^Easteal and Woolf;^66^

^*d*^Marcus and Hefter;^67^

^*e*^Chernyak;^68^

^*f*^Petrella and Sacco;^69^

^*g*^Naejus *et al.*;^70^

^*h*^Barthel *et al*.^71^

### 3.1 Data sources and selection criteria

This section details the criteria we used for selecting the values to analyse and to employ in eqn (3). The values of the standard molar volumes, *V*  *i*, are taken from Millero's review^59^ in the case of water (with the exception of thiocyanate, for which the conventional volume indicated by Marcus,^58^ Table 2, has been used), and from Marcus and Hefter's review^51^ in the case of non-aqueous solvents. Not all the *V*  *i* values of electrolytes in non-aqueous solvents have been experimentally determined (and reviewed), but, using an extra-thermodynamic assumption, Marcus and Hefter^51^ calculated the *V*  *i* values of the single ions in solution, which are additive. We have therefore calculated the missing values (where possible) using the ionic standard partial molar volumes. These values are affected by a significant error, ±2 cm^3^ mol^–1^ for the ion ⇒ ±4 cm^3^ mol^–1^ for the electrolyte, and the associated error bars are plotted in the standard and electrostrictive volume plots. The bars that have no error bars were calculated using the recommended, experimentally measured, (many independent sources obtained consistent results) standard partial molar volume values according to the reviews of Millero^59^ for water and Marcus and Hefter^51^ for non-aqueous solvents.

**Table 2:** Specific-ion series exhibited in the standard molar volume of electrolytes *V*  *i*: anions arranged by a common cation^*a*^

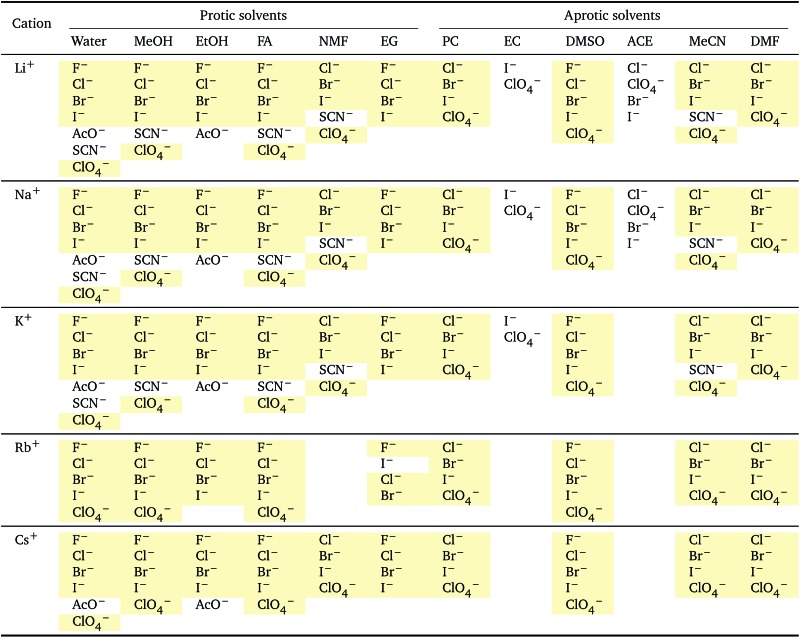

^*a*^For each cation, the anions are listed from smallest (top) to largest (bottom) standard molar volume. The cell background is coloured if that order of ions corresponds to a known specific-ion effects series. Pale yellow corresponds to a direct Hofmeister series.

The intrinsic molar volumes of the electrolytes, *V*_*i* intr_, cannot be experimentally measured. Several methods to estimate it have been proposed (for a review of them, see Marcus,^58^ Section 3.2), but no general agreement on the best method exists. Some of these methods rely on the ionic radii to estimate the intrinsic molar volume of an electrolyte, we think these values are sensitive to the assumptions of the model chosen so we do not follow this approach. According to Marcus,^58^ reasonable estimates (independent of the ionic radii) of the intrinsic molar volume of an electrolyte in solution are the ones proposed by Pedersen *et al.*,^72^ who extrapolated the intrinsic volume of the molten electrolyte down to room temperature (assuming the expansivity coefficient is constant), and by Marcus,^73^ who calculated the intrinsic volume for highly soluble salts, by extrapolating their partial molar volumes in concentrated solution up to a concentration where all the water present is completely electrostricted. If this information was not available, an acceptable estimation of the intrinsic molar volume was obtained from the crystal molar volume *V*_cryst_ (eqn (4)), which was first used by Padova:^74^

4

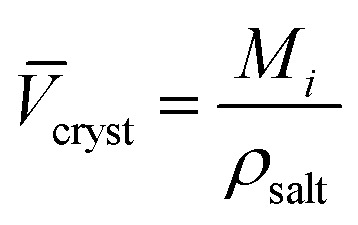

where *V*_cryst_ is the molar volume of the crystalline electrolyte, *M*_*i*_ the molar mass of the electrolyte and *ρ*_salt_ is the density of the salt. This method requires that the coordination number of the cations and anions is the same in the crystal lattice as in solution. This estimate is the most prone to error, as it is well known that coordination numbers are not the same for the same ion across different solvents.^75^

**Fig. 5:**
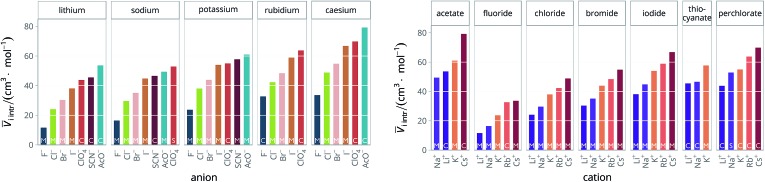
Intrinsic molar volumes *V*_i intr_ of the electrolytes grouped by cation (left) and by anion (right). The white labels inside the plot bars indicate the literature source. M: values calculated by Pedersen *et al.* ^72^ (extrapolation from molten salts); S: “soluble salts”, the estimates made by Marcus;^73^ C: crystal volume, as in eqn (4).

Therefore, for the purpose of this work, we used the estimates calculated by Pedersen *et al.* ^72^ when available, otherwise we used the ones given by Marcus in his 2010 paper,^73^ and *V*_cryst_ as the last resort. These criteria were used to determine *V*_*i* intr_ for all the electrolytes used in this work. The intrinsic molar volumes of the electrolytes used in this work are shown in Fig. 5.

The ionic intrinsic molar volumes are even more elusive quantities to calculate, as it requires again an extra-thermodynamic assumption to split the electrolytes volumes into their ionic constituents, thus introducing new sources of error. In addition, although ionic quantities are convenient, they do not reflect the physical reality as cations or anions are never on their own in solution. We therefore decided to restrict our analysis only to the electrolytes as a whole.

## 4 Results and discussion

### 4.1 Standard molar volumes

**Fig. 6:**
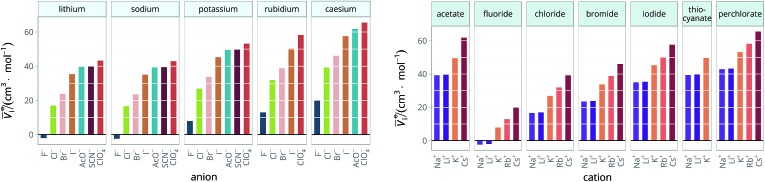
Standard molar volumes *V*  *i* of the electrolytes in water grouped by cation (left) and by anion (right).

The standard partial molar volumes are shown for water in Fig. 6. Note that all the values used in the calculations and the calculated values are provided in the ESI file, Table S1.† The standard partial molar volumes in all the remaining solvents are provided in the ESI file (Fig. S1 to S11†).

The series observed in each solvent for electrolytes sharing a common ion are outlined in Tables 2 and 3. In order to make the large amount of information in the table more accessible, the cells have been colour coded to indicate when the ordering of the ions corresponds to a series. The colour coding legend is given in the table caption. Where an overall series is found but an ion is incorrectly placed in the series, no colour is used for the incorrectly placed ion. It is apparent that the standard molar volumes follow the Hofmeister series for the anions, and the reverse lyotropic series for the cations. Note that as data is only available for two anions in ethylene carbonate, no series can be recognised but the ordering of the two ions is in line with the Hofmeister series and therefore consistent with the other solvents. The only exception observed for the anions is the ion-specific ordering observed in acetone, it follows an order that does not correspond to any known series. With regard to the cations, data is only available for two cations in acetone so no series can be recognised, but the ordering of the two ions is consistent and in line with the the reverse lyotropic series and therefore confers with the results for other solvents. An exception to the reverse lyotropic series observed is for cations paired with iodide in ethylene glycol. Additionally, the order of the sodium and lithium cations is reversed in water compared to all of the other solvents, indicating that for one of these ions there is a solvent interaction that is not seen in other solvents. It is evident from this evaluation of the standard molar volumes of electrolytes in a wide range of solvents that we can order the specific-ion effects into a fundamental series that is independent of the solvent. Moreover standard molar volume is a bulk property. This infers that the series arises solely from the properties of the ions and is almost completely independent of the solvent and extant at low concentration and in the absence of surfaces. We discuss the implications of this discovery further below.

**Table 3:** Specific-ion series exhibited in the standard molar volume of electrolytes *V*  *i*: cations arranged by a common anion^*a*^

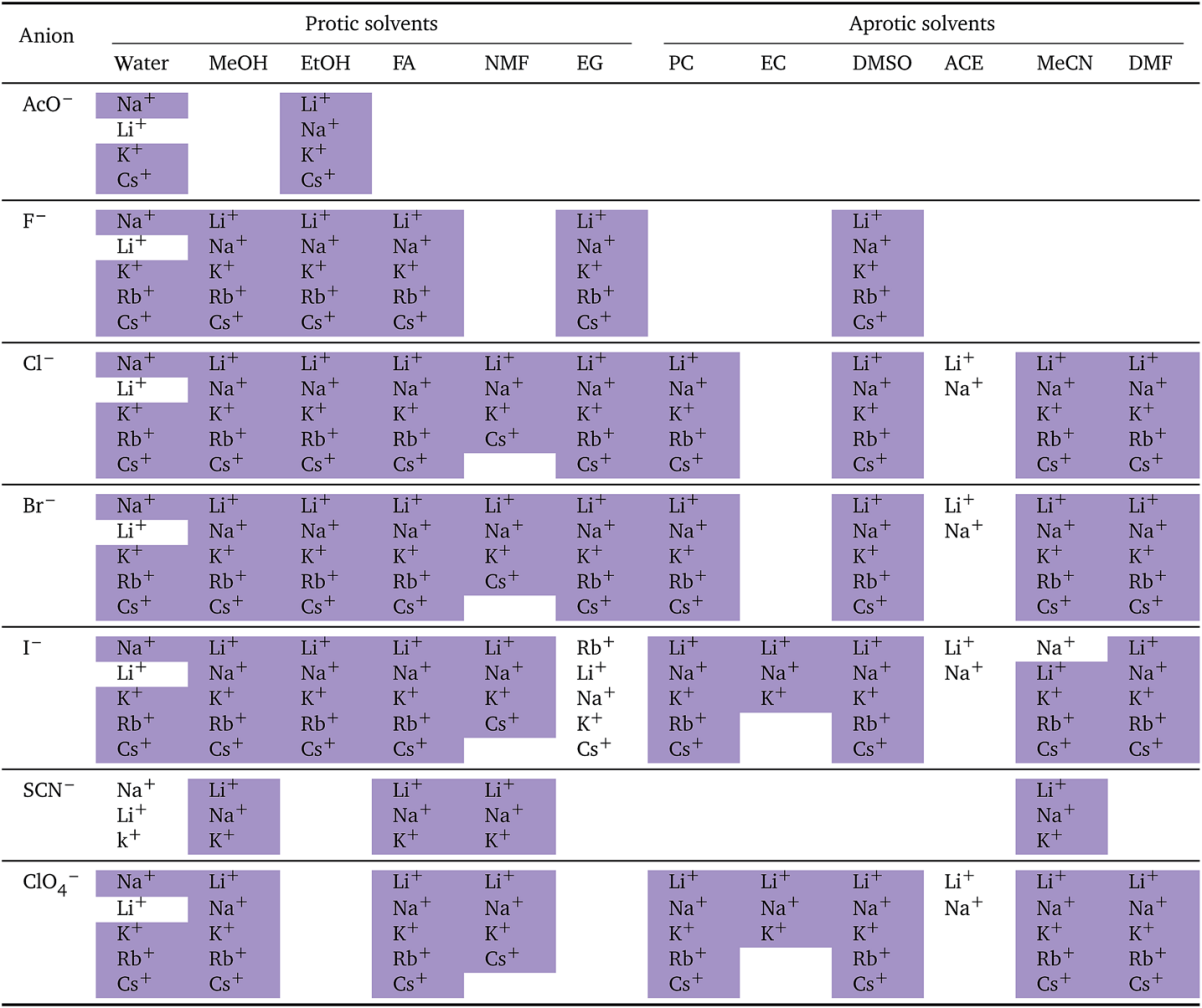

^*a*^For each anion, the cations are listed from smallest (top) to largest (bottom) standard molar volume. The cell background is coloured if that order of ions corresponds to a known specific-ion effects series. Purple corresponds to a reverse lyotropic series.

### 4.2 Electrostriction in water and non-aqueous solvents

Specific-ion effects are often attributed to the influence that the ions exert on the solvent, most often water. This has led to terms such as structure-making/structure-breaking and chaotropic and kosmotropic. These arguments generally follow a consideration of the size of an ion and its electrostatic charge. The charge on an ion produces a large electric field and this exerts a pressure on the surrounding solvent molecules that results in electrostriction. Therefore, we have also explored electrostriction as a measure of ion specificity in a wide range of solvents.

**Fig. 7:**
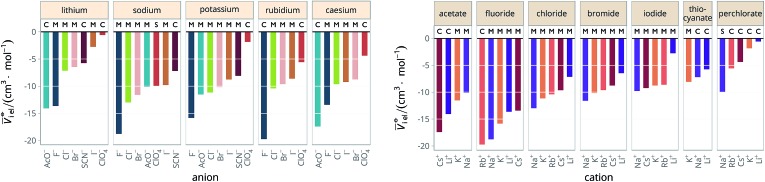
Molar electrostrictive volume *V*  *i* el of alkali metal salts in water at infinite dilution grouped by cation (left) and by anion (right).

The molar electrostrictive volumes that we have calculated from eqn (3) for water are shown in Fig. 7. The electrostrictive volumes in all the remaining solvents are provided in the ESI file (Fig. S12 to S22†). We first note that the electrostrictive volumes for ions sharing a common counterion are different: the magnitude of electrostriction is therefore ion-specific in all of the solvents considered. The series observed in each solvent for electrolytes sharing a common ion are outlined in Tables 4 and 5. For the anion series (shown in Table 4), surprisingly consistent behaviour is observed across the different solvents. The trend observed is predominantly a Hofmeister series across all protic and aprotic solvents. The exceptions are: a reversal of the series in ethanol, a reversal of the series for the caesium salts in *N*-methylformamide and a lyotropic series for the sodium salts in water. The assignment of the series for the sodium salts in water is equivocal, as the electrostrictive values for sodium perchlorate and sodium iodide are very close. If their order is reversed, this system is also consistent with the Hofmeister series. No known ion-specific ordering is observed in acetone, for sodium salts in methanol, for lithium and caesium salts in ethanol, and for lithium salts in formamide. The preponderance of the Hofmeister series produced from the electrostrictive volumes for anions across a wide range of solvents is in accordance with the domination of the Hofmeister series for anions derived from the standard molar volumes, adding weight to the argument that the anions fundamentally give rise to a Hofmeister series independent of the solvent.

**Table 4:** Specific-ion series exhibited in the electrostrictive volume of electrolytes *V*  *i* el: anions arranged by a common cation^*a*^

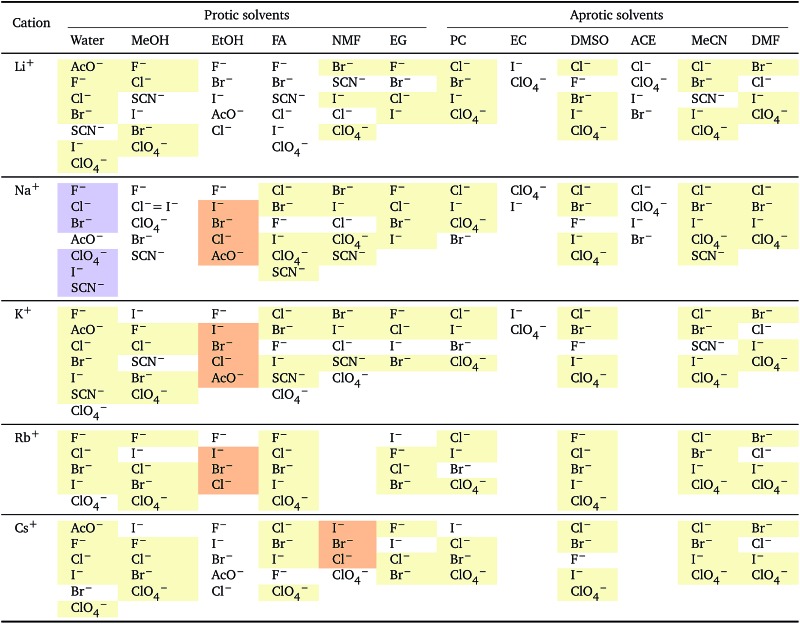

^*a*^For each cation, the anions are listed from largest (top) to smallest (bottom) electrostrictive volume. The cell background is coloured as indicated in the legend if that order of ions corresponds to a known specific-ion effects series.
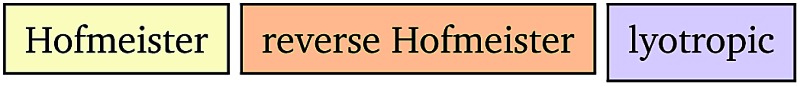

**Table 5:** Specific-ion series exhibited in the electrostrictive volume of electrolytes *V*  *i* el: cations arranged by a common anion^*a*^

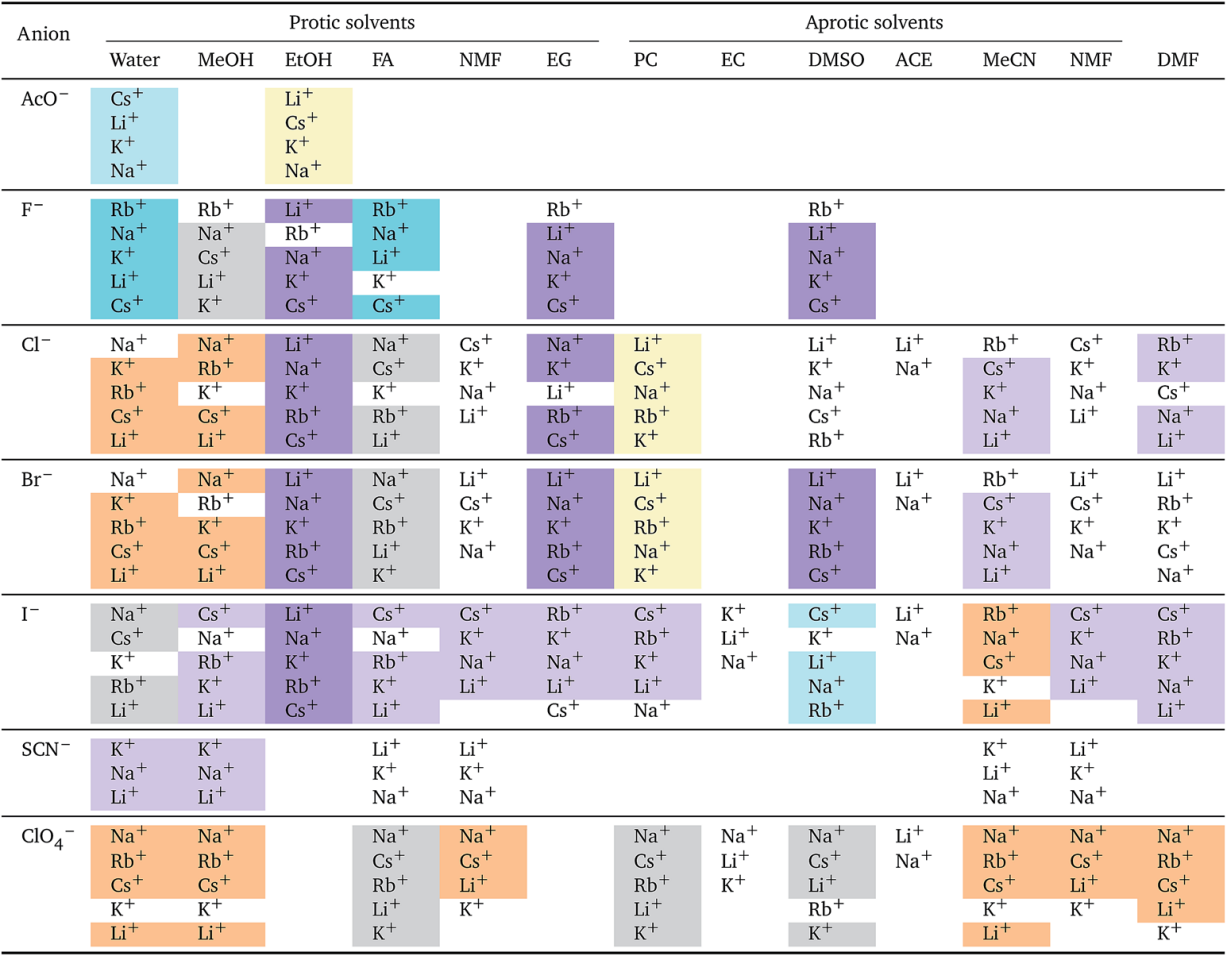

^*a*^For each anion, the cations are listed from largest (top) to smallest (bottom) electrostrictive volume. The cell background is coloured as indicated in the legend if that order of ions corresponds to a known specific-ion effects series. 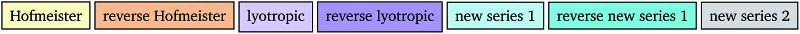

The situation for the cations is very different (Table 5). All the known series plus an additional two are observed. Although the lyotropic series (forward or reverse) is the most common, the variety of behaviour is large and does not seem to correlate to any property of the solvent or the counterion. The difference with respect to Table 4 is striking. It has to be noted that the differences in electrostriction values across cations are much smaller than those for anions and therefore the uncertainty in the estimate of the standard molar volume is more significant.

The wide range of behaviour seen reflects the complexity we have observed in measurements of limiting molar conductivity and viscosity *B*-coefficients across a range of solvents reported in our earlier work.^23^

### 4.3 Electrostriction normalised by electrolyte size

The electrostriction values above are strongly influenced by the size of the ions themselves. We wish to evaluate the degree of electrostriction relative to the size of the ion. This can be done by normalising the electrostrictive volumes calculated above by the intrinsic molar volume of the electrolyte in that solvent: in this way, the relative degree of electrostriction compared to the ion size is evaluated.

We therefore performed the following calculation:
5

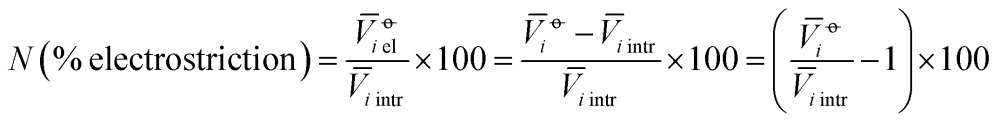

where *V*  *i* is the standard partial molar volume of the species “*i*” in solution, and *V*_*i* intr_ is the intrinsic molar volume of the species “*i*”.

**Fig. 8:**
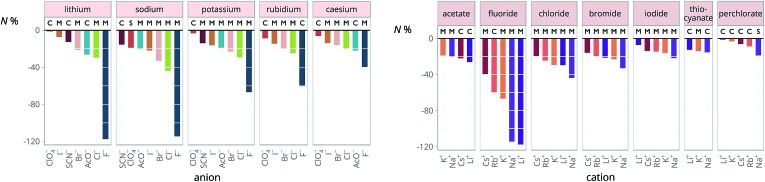
Normalised molar electrostrictive volume *N*% of alkali metal salts in water at infinite dilution grouped by cation (left) and by anion (right).

The new quantity *N* is dimensionless and represents a “normalised” electrostriction under the condition that all the electrolytes had the same intrinsic molar volume (without accounting for changes in charge density, polarisability, *etc.*). This quantity is equal to 0 when the intrinsic and standard volumes are exactly the same (*i.e.* no electrostriction of the solvent has happened, as in case I of Fig. 3); negative when electrostriction takes place: the larger the electrostriction, the more negative the normalised quantity becomes. Two sub-cases can be identified when *N* is negative: –100% < *N* < 0 when the molar volume of the electrolyte is positive (case II of Fig. 3), whereas *N* < –100% for case III, when the molar volume of the electrolyte is smaller than 0 and therefore the final solution volume is even smaller than the original pure solvent volume. Finally, *N* is positive when expansion of the solvent happens as in case IV of Fig. 3. The resulting plots for electrolytes in water are in Fig. 8. The plots for all the remaining solvents are in the ESI file (Fig. S23 to S33†). Tables 6 and 7 summarise the series observed in the different solvents, grouping electrolytes by cation and anion, respectively. The effect of the normalisation is an even more homogeneous picture for the anion behaviour (Tables 6 *versus* 4), where the Hofmeister series is nearly universal, with the only exception being lithium salts in acetone.

**Table 6:** Specific-ion series exhibited in the normalised electrostrictive volume of electrolytes, *N*%: anions arranged by a common cation^*a*^

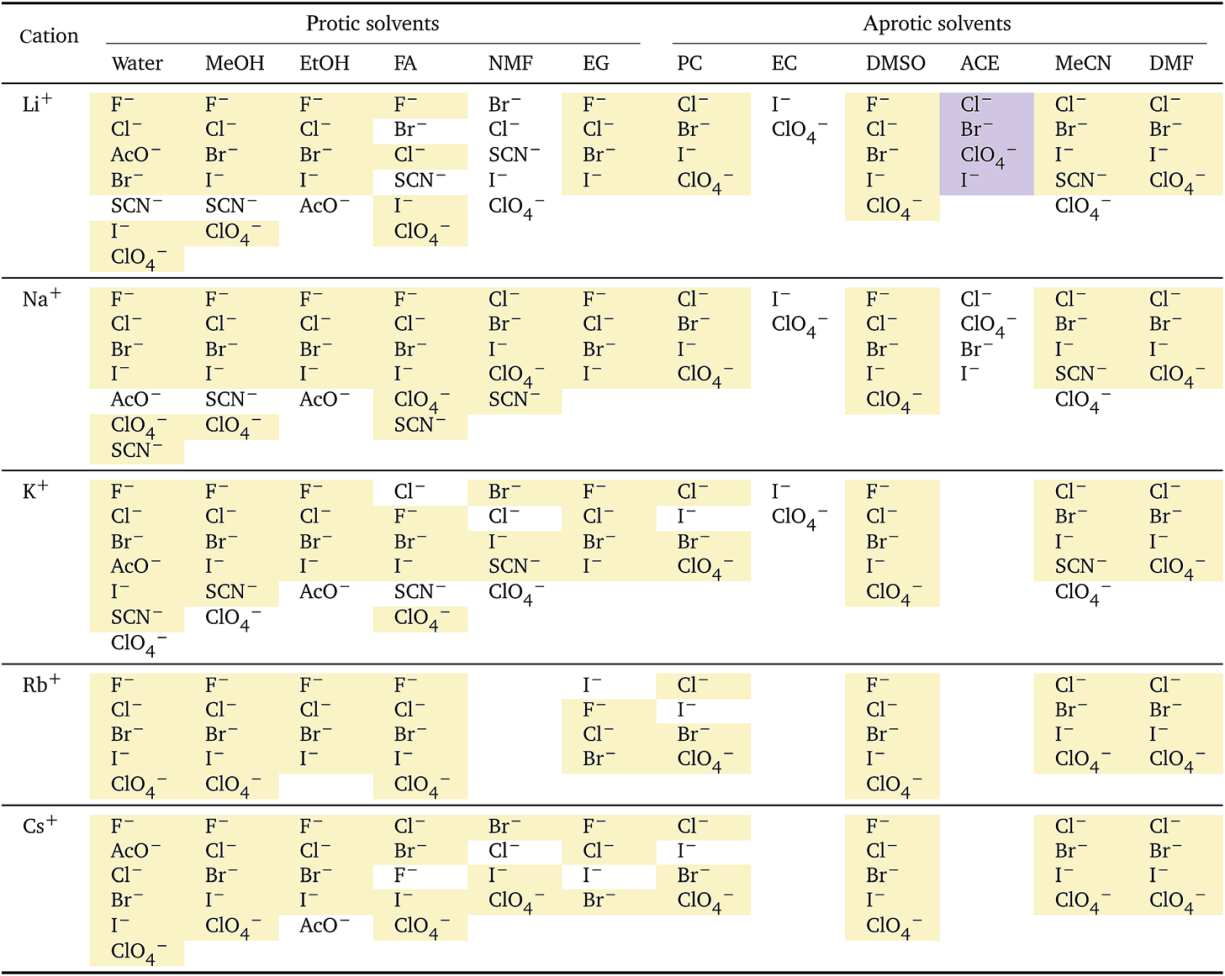

^*a*^For each cation, the anions are listed from largest (top) to smallest (bottom) normalised electrostrictive volume. The cell background is coloured as indicated in the legend if that order of ions corresponds to a known specific-ion effects series.
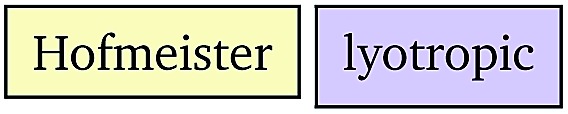

**Table 7:** Specific-ion series exhibited in the normalised electrostrictive volume of electrolytes, *N*%: cations arranged by a common anion^*a*^

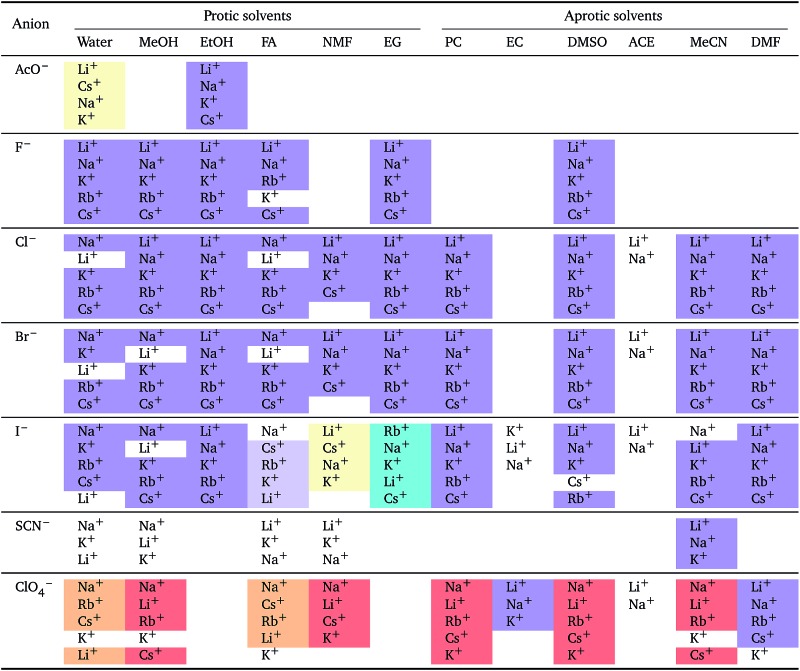

^*a*^For each anion, the cations are listed from largest (top) to smallest (bottom) normalised electrostrictive volume. The cell background is coloured as indicated in the legend if that order of ions corresponds to a known specific-ion effects series.
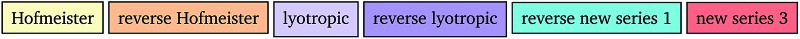

Significantly for the cations shown in Table 7: the reverse lyotropic series is preponderant, as it was for the standard partial molar volumes (Table 2). The most variability in behaviour across solvents is observed for polyatomic ions, for which the Hofmeister series and reverse Hofmeister series are observed and an altogether new series occurs as well. Polyatomic ions have permanent multipole moments due to the aspherical distribution of charge, therefore it is unsurprising that electrostriction does not follow the same series evident for the monoatomic anions. If the polyatomic anions are set aside, the reverse lyotropic series is nearly universal. This is a dramatic change from the prenormalised electrostrictive volumes summarised in Table 5. The preponderance of the reverse lyotropic series produced from the normalised electrostrictive volumes for cations across a wide range of solvents is in accordance with the domination of the reversed lyotropic series for anions derived from the standard molar volumes, adding weight to the argument that the cations fundamentally give rise to a reversed lyotropic series independent of the solvent.

### 4.4 General implications

From this work we can draw a number of conclusions regarding specific-ion effects.

We find that the ordering of specific-ion effects for the standard molar volumes and the normalised electrostriction of electrolytes at infinite dilution is independent of the solvent. This ordering follows a Hofmeister series for anions and a reversed lyotropic series for cations, which correspond to smaller “harder” cations and anions having a smaller intrinsic volume *V*  *i* and causing a larger normalised electrostriction *N*% than large, “soft” cations and anions. As a consequence, the grouping usually associated with the Hofmeister series is respected for anions. That is the “structure makers” or “kosmotropes” which are ions small in size and with high surface charge density and small polarisability give rise to the largest electrostriction. This does not hold for the cations, where the Hofmeister series ordering is lost and an ordering that follows the reverse lyotropic series which is determined by the cation size is instead in place.

This solvent-independence of electrostriction in the limit of infinite dilution justifies a theoretical continuum approach for systems respecting this constraint (it remains to be understood when this condition is no longer valid). This demonstrates that, at a fundamental level, such as for bulk properties of very diluted solutions, the ordering of specific-ion effects are solely a property of the ions and the only essential ingredient for specific-ion effects to happen is the presence of electrolytes. We note that most experimental observations of specific-ion effects are made at moderately high to high electrolyte concentrations. This may lead to the erroneous conclusion that these effects are only manifest at high concentrations. In fact neither surfaces nor higher concentrations are needed. Of course the solvents do matter in that they mediate the strength of the effects. This finding is corroborated by the trends that we found previously^23^ for bulk properties such as the limiting molar conductivity and the viscosity *B*-coefficients, where a lyotropic series is predominant for the alkali metals cations and a Hofmeister series for the anions. Whereas other experiments involving ions at high concentration and surfaces result in a perturbation to the fundamental series observed here.^23^ These perturbations likely arise due to a complex interaction of ion pairing, dispersion forces, and solvation both in the bulk and at interfaces. Furthermore, the balance of these effects will depend on temperature as well as the specifics of the solvent and surfaces.^76^ We believe that an improved understanding of the suite of specific-ion effects observed may be gained by considering them as perturbations to the fundamental series observed here in the simplest systems.

The homogeneity in behaviour of electrostrictive phenomena must not shadow the fact that ion solvation mechanisms differ greatly across solvents. Electrostriction is only one consequence of ion solvation and does not capture the local ion–solvent interactions or the structure of the solvent around solvated ions. The chemical properties, molecular size and polarisability of the solvents vary widely. The interactions between ions and solvent are reflected in selective ion–solvent interactions. These have been shown to vary greatly for solvents of different polarisability, leading to different trends in solvation.^77,78^ The thermodynamics of solvation across solvents and the effect of ions on solvent structure and on solvent dynamics needs to be considered to obtain a comprehensive physical picture. We are addressing some of these matters in a forthcoming paper.

How might this information be useful in furthering our understanding of specific-ion effects? This analysis tells us that calculations and simulations of specific-ion effects in dilute solutions should be able to reproduce the Hofmeister series for anions and the lyotropic series for cations without specifically accounting for the solvent, supporting an approach already being employed.^79^ The details of the solvent will matter in determining the magnitude of the effect. Our interpretation is that, when the system becomes more complex, for instance because a surface comes into play, or because the electrolyte concentration is increased, those fundamental specific-ion effects are perturbed and both surfaces and the solvent come into play and determine their final ordering.

## 5 Conclusions

We have analysed the trends of specific-ion effects for standard molar volumes and standard electrostrictive volumes. We have performed a normalisation of the electrostrictive volumes in order to compare across ions and solvents. Our work evidences the presence of specific-ion effects at low concentration, a notion that has often been disregarded in the literature, where many authors associate specific-ion effects only with higher salt concentrations.^80^ This is perhaps due to the magnitude of these effects being small for many measurements at low concentration, and in some systems specific-ion effects may only be revealed once electrostatic effects have been quenched by screening.

Electrostriction is an ion-specific effect in all of the electrolyte solutions considered. This confirms that, with respect to specific-ion effects, water is not a “special” solvent. This also rejects the idea of an exclusive association between the existence of specific-ion effects and the presence of a hydrogen-bonding network: aprotic solvents show ion specificity as well, indeed ions in all solvents show ion specificity. Further, the complexity conferred by ion specificity is necessary for life, but ion specificity is not restricted to aqueous solutions.

Our findings support the hypothesis that the ordering of specific-ion effects is fundamentally due to properties of the ions themselves and that this fundamental ordering is exhibited experimentally under appropriate circumstances. This is exemplified by the partial molar volume and normalised electrostriction of anions following a Hofmeister series whilst the cations follow a reverse lyotropic series. The quantitative connection between these series and the individual properties of the ions is not trivial and remains the realm of active efforts in this field. This outcome is important for our understanding of specific-ion effects. In models of bulk properties of solvents in the limit of infinite dilution, the specific nature or the granularity of the solvent should not determine the order of the specific-ion effects.

## Conflicts of interest

The authors have no conflicts to declare.
