# Correction: What is the fundamental ion-specific series for anions and cations? Ion specificity in standard partial molar volumes of electrolytes and electrostriction in water and non-aqueous solvents

**DOI:** 10.1039/c9sc90050k

**Published:** 2019-03-04

**Authors:** Virginia Mazzini, Vincent S. J. Craig

**Affiliations:** a Department of Applied Mathematics , Research School of Physics and Engineering , The Australian National University , Canberra , ACT 2601 , Australia . Email: vince.craig@anu.edu.au

## Abstract

Correction for ‘What is the fundamental ion-specific series for anions and cations? Ion specificity in standard partial molar volumes of electrolytes and electrostriction in water and non-aqueous solvents’ by Virginia Mazzini *et al.*, *Chem. Sci.*, 2017, **8**, 7052–7065.



The authors regret that Fig. 1 is incorrect in the original article. The correct version of Fig. 1 is presented below.

The “forward” direction of the alkali metal cations, bivalent cations and guanidinium ion is now reversed, with the most effective cations at precipitating proteins now correctly indicated. The relative positioning of the tetraalkylammonium ions with respect to the alkali metal cations is also corrected (their “forward” direction was correct in Fig. 1 in the original article and need not be reversed). The lyotropic series of cations has been flipped, so that it runs in the same direction as the cation Hofmeister series.

**Fig. 1:**
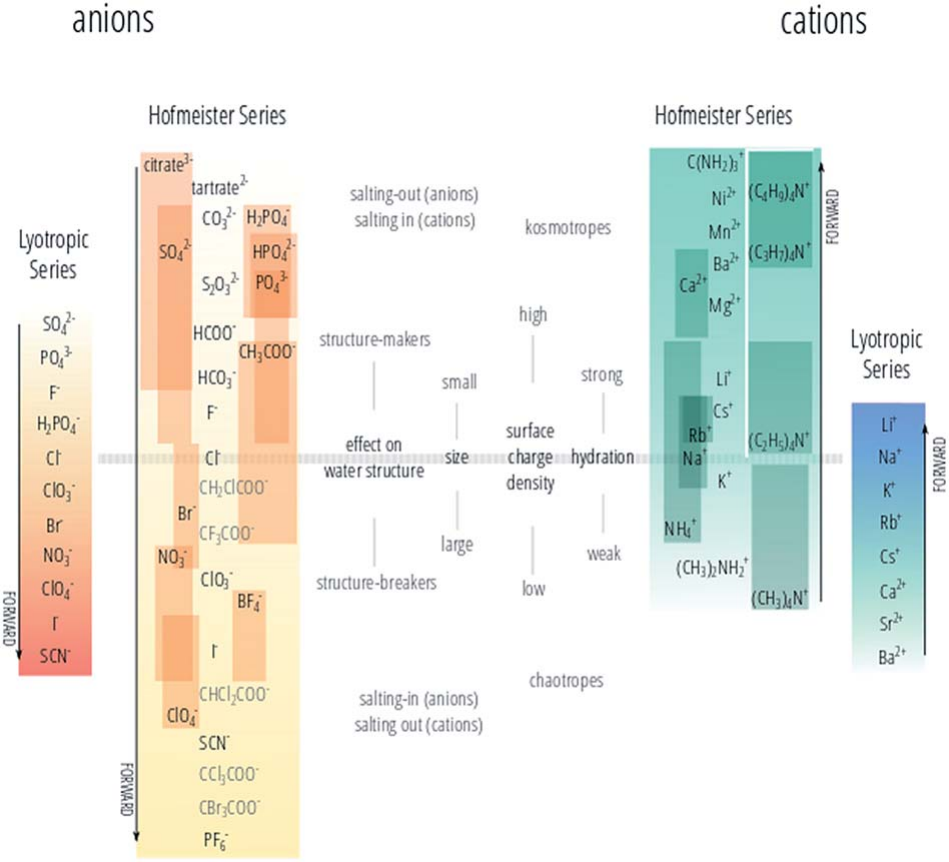
The Hofmeister and lyotropic series of ions in water. The forward direction for each series is indicated by the corresponding arrow. For the Hofmeister ions the forward series is the one of decreasing effectiveness at precipitating proteins out of solution. For the lyotropic ions, the forward series corresponds to increasing lyotropic number. The Hofmeister series for anions was obtained by combining the series reported in ref. 9, 12, and 24–33 in the original article. The Hofmeister series for cations was obtained by combining the series reported in ref. 25, 29–31 and 34–36 in the original article and ref. 1 herein. The ion is positioned in its most agreed upon ranking, and bars indicate the variations in position among different publications. The relative positioning of the haloacetates is well-known, but their positioning with respect to the “classic” anions of the series is certain only in a few cases. This uncertainty is reflected by presenting these ions in grey text rather than black. The ethyl- to butylammonium cations ordering is known with respect to the tetramethylammonium ion, but not to the other cations in the series with certainty. A white line has therefore been drawn around these ions to mark the discontinuity. Ions at one end of the series are often attributed with having the opposite effect to ions at the other end of the series. As such there is a point in the series where the influence of ions reverses. The grey horizontal line traces the divide that corresponds to this property reversal. Exceptions to this classification are Rb^+^ and Cs^+^, which are larger and less hydrated than Na^+^ and K^+^; the guanidinium ion (C(NH_2_)_3_^+^) and the tetraalkylammonium cations. The ions of the lyotropic series, as defined by Voet (ref. 18 in the original article) are integrated with those from Marcus (ref. 49 in the original article) and their “forward” direction corresponds to increasing lyotropic number.

The authors also regret that Tables 5 and 7 are incorrect. The correct versions of Tables 5 and 7 are presented below.

In both tables, the “forward” Hofmeister series (light yellow background) has been corrected to the “reverse” Hofmeister series (light orange background). In Table 5, the “NMF” column under “Aprotic solvents” was an error and has been removed.

**Table 1:** Specific-ion series exhibited in the electrostrictive volume of electrolytes *V[combining macron]* <svg xmlns="http://www.w3.org/2000/svg" version="1.0" width="16.000000pt" height="16.000000pt" viewBox="0 0 16.000000 16.000000" preserveAspectRatio="xMidYMid meet"><metadata> Created by potrace 1.16, written by Peter Selinger 2001-2019 </metadata><g transform="translate(1.000000,15.000000) scale(0.005147,-0.005147)" fill="currentColor" stroke="none"><path d="M960 2600 l0 -120 -120 0 -120 0 0 -80 0 -80 -80 0 -80 0 0 -120 0 -120 -200 0 -200 0 0 -80 0 -80 200 0 200 0 0 -120 0 -120 80 0 80 0 0 -120 0 -120 120 0 120 0 0 -80 0 -80 320 0 320 0 0 80 0 80 80 0 80 0 0 120 0 120 120 0 120 0 0 120 0 120 200 0 200 0 0 80 0 80 -200 0 -200 0 0 120 0 120 -120 0 -120 0 0 80 0 80 -80 0 -80 0 0 120 0 120 -320 0 -320 0 0 -120z m640 -200 l0 -80 80 0 80 0 0 -120 0 -120 -520 0 -520 0 0 120 0 120 120 0 120 0 0 80 0 80 320 0 320 0 0 -80z m160 -600 l0 -120 -80 0 -80 0 0 -120 0 -120 -320 0 -320 0 0 120 0 120 -120 0 -120 0 0 120 0 120 520 0 520 0 0 -120z"/></g></svg> *i* el: cations arranged by a common anion^*a*^

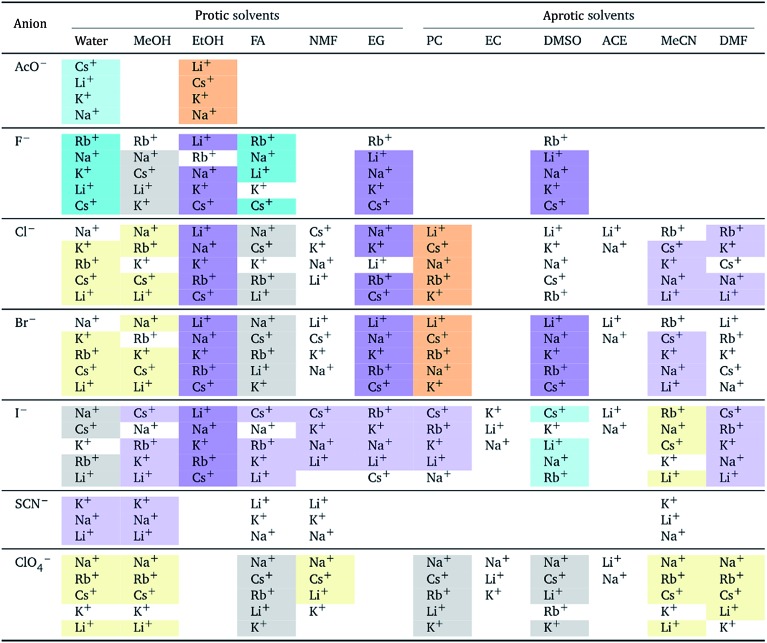

^*a*^For each anion, the cations are listed from largest (top) to smallest (bottom) electrostrictive volume. The cell background is coloured as indicated in the legend if that order of ions corresponds to a known specific-ion effects series.
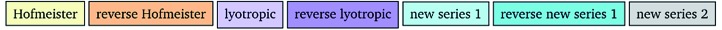

**Table 2:** Specific-ion series exhibited in the normalised electrostrictive volume of electrolytes, *N*%: cations arranged by a common anion^*a*^

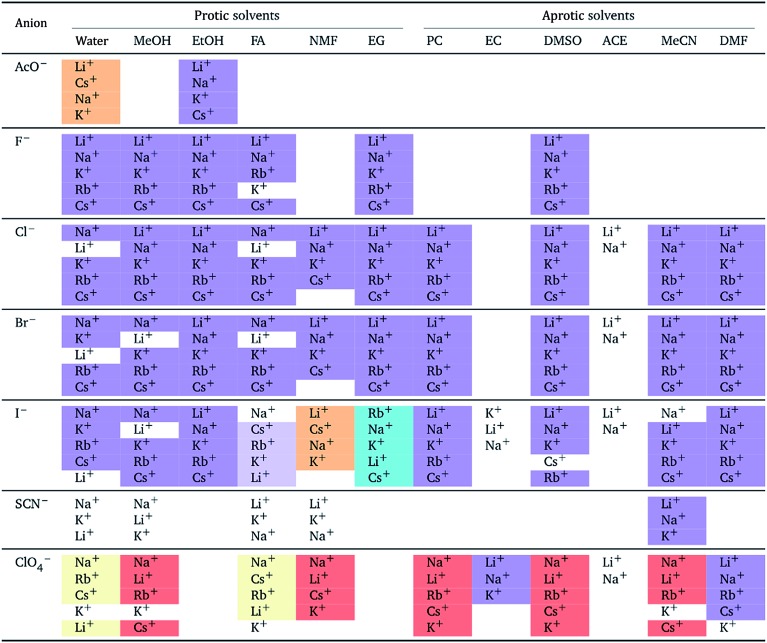

^*a*^For each anion, the cations are listed from largest (top) to smallest (bottom) normalised electrostrictive volume. The cell background is coloured as indicated in the legend if that order of ions corresponds to a known specific-ion effects series.
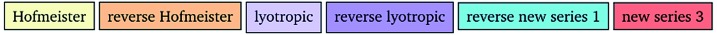

The Royal Society of Chemistry apologises for these errors and any consequent inconvenience to authors and readers.
