# Supplementary material for: What is the fundamental ion-specific series for anions and cations? Ion specificity in standard partial molar volumes of electrolytes and electrostriction in water and non-aqueous solvents
Source: Chem Sci. 2017 Aug 21;8(10):7052–65. doi: 10.1039/c7sc02691a (PMC5637464; doi:10.1039/c7sc02691a)
Supplement: Supplementary file 1 [file SC-008-C7SC02691A-s001.pdf]

Electronic Supplementary Material (ESI) for Chemical Science.
This journal is © The Royal Society of Chemistry 2017

Electronic Supplementary Information

What is the fundamental ion specific series for anions and cations? Ion specificity in standard partial molar volumes of electrolytes and electrostriction in water and non-aqueous solvents

Virginia Mazzini[a] and Vincent S. J. Craig[a,*]

[a] Department of Applied Mathematics, Research School of Physics and Engineering, The Australian National University, Canberra, ACT, 2601, Australia.
[*] corresponding author, E-mail: vince.craig@anu.edu.au

Contents

Tabulation of the volume values S2

standard molar volumes of electrolytes S7
protic solvents S7
aprotic solvents S8

electrostrictive volume of electrolytes S10
protic solvents S10
aprotic solvents S11

normalised electrostrictive volume of electrolytes S13
protic solvents S13
aprotic solvents S14

Tabulation of the volume values

Table S1 Values of all the volumes used in this paper

electrolyte	solvent	$\overline{V}_{\mathrm{i\ intr}}$	mode[a]	$\overline{V}_{\mathrm{i}}^{\ominus}$	minimum error[b]	$\overline{V}_{\mathrm{i\ el}}^{\ominus}$	N(% electrostr)
RbF	water	32.6	C	12.9	R	−19.7	−60.5
NaF	water	16.4	M	−2.4	R	−18.8	−114.5
CsOAc	water	79.2	C[d]	61.8	R	−17.4	−22.0
KF	water	23.7	M	7.9	R	−15.8	−66.8
LiOAc	water	53.6	C[c]	39.6	R	−14.1	−26.2
LiF	water	11.6	M	−2.0	R	−13.6	−117.6
CsF	water	33.6	M	20.2	R	−13.4	−39.9
NaCl	water	29.6	M	16.6	R	−13.0	−43.9
NaBr	water	35.1	M	23.5	R	−11.6	−33.0
KOAc	water	61	S	49.5	R	−11.5	−18.9
KCl	water	38	M	26.9	R	−11.2	−29.3
RbCl	water	42.3	M	31.9	R	−10.4	−24.6
KBr	water	43.9	M	33.7	R	−10.2	−23.2
NaOAc	water	49.4	S	39.3	R	−10.2	−20.5
$NaClO_4$	water	52.9	S	42.9	R	−10.0	−18.9
NaI	water	44.8	M	35.0	R	−9.8	−21.9
CsCl	water	48.8	M	39.2	R	−9.6	−19.7
RbBr	water	48.4	M	38.8	R	−9.6	−19.9
CsI	water	66.8	M	57.6	R	−9.2	−13.8
KI	water	54	M	45.2	R	−8.8	−16.2
CsBr	water	54.8	M	46.1	R	−8.8	−16.0
RbI	water	58.9	M	50.3	R	−8.6	−14.6
KSCN	water	57.7	S	49.6	R	−8.1	−14.0
NaSCN	water	46.6	C[f]	39.4	R	−7.2	−15.5
LiCl	water	24.1	M	17.0	R	−7.2	−29.7
LiBr	water	30.3	M	23.8	R	−6.5	−21.4
LiSCN	water	45.5	C[e]	39.7	R	−5.8	−12.6
$RbClO_4$	water	63.8	C	58.2	R	−5.6	−8.7
$CsClO_4$	water	69.8	C	65.5	R	−4.4	−6.3
LiI	water	38.1	M	35.3	R	−2.8	−7.2
$KClO_4$	water	55.0	C	53.1	R	−1.8	−3.3
$LiClO_4$	water	43.8	C	43.2	R	−0.6	−1.3
RbF	MeOH	32.6	C	−5	4	−37.6	−115.3
NaF	MeOH	16.4	M	−20	4	−36.4	−222.0
CsI	MeOH	66.8	M	33	4	−33.8	−50.6
NaCl	MeOH	29.6	M	−3.9	R	−33.5	−113.2
NaI	MeOH	44.8	M	11.3	R	−33.5	−74.8
RbI	MeOH	58.9	M	26	4	−32.9	−55.9
KI	MeOH	54	M	21.7	R	−32.3	−59.8
CsF	MeOH	33.6	M	2	4	−31.6	−94.0
LiF	MeOH	11.6	M	−20	4	−31.6	−272.4
KF	MeOH	23.7	M	−7.6	R	−31.3	−132.1
RbCl	MeOH	42.3	M	11	4	−31.3	−74.0
KCl	MeOH	38	M	7	R	−31	−81.6
$NaClO_4$	MeOH	52.9	S	22	4	−30.9	−58.4
NaBr	MeOH	35.1	M	4.3	R	−30.8	−87.7
CsCl	MeOH	48.8	M	18	4	−30.8	−63.1
KSCN	MeOH	57.7	S	28	4	−29.7	−51.5
NaSCN	MeOH	46.6	C[f]	17	4	−29.6	−63.5
RbBr	MeOH	48.4	M	19	4	−29.4	−60.7
KBr	MeOH	43.9	M	14.7	R	−29.2	−66.5
CsBr	MeOH	54.8	M	26	4	−28.8	−52.6

Volumes and error in $cm^3\,mol^{-1}$.
[a]M: from molten salts[S1]; S: from soluble salts[S2]; C: calculated from density of the crystal[S3]; [b] "R" indicates a value recommended by the reviewer[S4,S5]. [c] from Ref. S6; [d] Ref. S7; [e] Ref. S8; [f] Ref. S9.

electrolyte	solvent	$\overline{V}_{\mathrm{i\ intr}}$	mode[a]	$\overline{V}_{\mathrm{i}}^{\ominus}$	minimum error[b]	$\overline{V}_{\mathrm{i\ el}}^{\ominus}$	N(% electrostr)
LiCl	MeOH	24.1	M	−4.5	R	−28.6	−118.7
LiSCN	MeOH	45.5	C[e]	17	4	−28.5	−62.6
LiI	MeOH	38.1	M	11	4	−27.1	−71.1
$RbClO_4$	MeOH	63.8	C	37	4	−26.8	−42.0
LiBr	MeOH	30.3	M	4	4	−26.3	−86.8
$CsClO_4$	MeOH	69.8	C	44	4	−25.8	−37.0
$KClO_4$	MeOH	55.0	C	33	4	−22.0	−40.0
$LiClO_4$	MeOH	43.8	C	22	4	−21.8	−49.8
LiF	EtOH	11.6	M	−35	4	−46.6	−401.7
RbF	EtOH	32.6	C	−8	4	−40.6	−124.5
NaF	EtOH	16.4	M	−23	4	−39.4	−240.2
KF	EtOH	23.7	M	−14	4	−37.7	−159.1
LiBr	EtOH	30.3	M	−5.2	R	−35.5	−117.2
CsF	EtOH	33.6	M	0	4	−33.6	−100
LiI	EtOH	38.1	M	5	4	−33.1	−86.9
LiOAc	EtOH	53.6	C[c]	22	4	−31.6	−59.0
LiCl	EtOH	24.1	M	−4.9	R	−29	−120.3
NaI	EtOH	44.8	M	16.2	R	−28.6	−63.8
KI	EtOH	54	M	25.5	R	−28.5	−52.8
RbI	EtOH	58.9	M	32	4	−26.9	−45.7
CsI	EtOH	66.8	M	40	4	−26.8	−40.1
NaBr	EtOH	35.1	M	9	4	−26.1	−74.4
KBr	EtOH	43.9	M	18	4	−25.9	−59.0
NaCl	EtOH	29.6	M	5	4	−24.6	−83.1
RbBr	EtOH	48.4	M	24	4	−24.4	−50.4
KCl	EtOH	38	M	14	4	−24	−63.2
CsBr	EtOH	54.8	M	32	4	−22.8	−41.6
RbCl	EtOH	42.3	M	20	4	−22.3	−52.7
CsOAc	EtOH	79.2	C[d]	57	4	−22.2	−28.0
CsCl	EtOH	48.8	M	28	4	−20.8	−42.6
KOAc	EtOH	61	S	43	4	−18	−29.5
NaOAc	EtOH	49.4	S	34	4	−15.4	−31.2
RbF	FA	32.6	C	24	4	−8.6	−26.5
NaCl	FA	29.6	M	21.3	R	−8.3	−28.0
NaBr	FA	35.1	M	28.2	R	−6.9	−19.7
CsCl	FA	48.8	M	42.3	R	−6.5	−13.3
NaF	FA	16.4	M	10	4	−6.4	−39.0
KCl	FA	38	M	31.7	R	−6.3	−16.6
RbCl	FA	42.3	M	36	4	−6.3	−14.9
CsBr	FA	54.8	M	48.9	R	−5.9	−10.8
CsI	FA	66.8	M	61	4	−5.8	−8.7
LiF	FA	11.6	M	6	4	−5.6	−48.3
RbBr	FA	48.4	M	43	4	−5.4	−11.2
LiBr	FA	30.3	M	25	4	−5.3	−17.5
KBr	FA	43.9	M	38.8	R	−5.1	−11.6
NaI	FA	44.8	M	40	R	−4.8	−10.7
LiSCN	FA	45.5	C[e]	41	4	−4.5	−9.8
LiCl	FA	24.1	M	19.9	R	−4.2	−17.4
RbI	FA	58.9	M	55	4	−3.9	−6.6
KF	FA	23.7	M	20	4	−3.7	−15.6
CsF	FA	33.6	M	30	4	−3.6	−10.7
KI	FA	54	M	50.6	R	−3.4	−6.3
KSCN	FA	57.7	S	55	4	−2.7	−4.7
$NaClO_4$	FA	52.9	S	51	4	−1.9	−3.6
NaSCN	FA	46.6	C[f]	45	4	−1.6	−3.4
LiI	FA	38.1	M	37	4	−1.1	−2.9

Volumes and error in $\mathrm{cm^3\,mol^{-1}}$.
[a]M: from molten salts[S1]; S: from soluble salts[S2]; C: calculated from density of the crystal[S3]; [b] "R" indicates a value recommended by the reviewer[S4,S5]. [c] from Ref. S6; [d] Ref. S7; [e] Ref. S8; [f] Ref. S9.

electrolyte	solvent	$\overline{V}_{\mathrm{i\ intr}}$	mode[a]	$\overline{V}^{\ominus}_{\mathrm{i}}$	minimum error[b]	$\overline{V}^{\ominus}_{\mathrm{i\ el}}$	N(% electrostr)
$CsClO_4$	FA	69.8	C	71	4	1.2	1.7
$RbClO_4$	FA	63.8	C	65	4	1.2	1.9
$LiClO_4$	FA	43.8	C	47	4	3.2	7.3
$KClO_4$	FA	55.0	C	61	4	6.0	10.9
RbI	EG	58.9	M	26	R	−32.9	−55.9
RbF	EG	32.6	C	17	4	−15.6	−47.9
LiF	EG	11.6	M	−3	4	−14.6	−125.9
NaF	EG	16.4	M	2	4	−14.4	−87.8
KF	EG	23.7	M	11	4	−12.7	−53.6
NaCl	EG	29.6	M	20.9	R	−8.7	−29.4
LiBr	EG	30.3	M	22.2	R	−8.1	−26.7
KCl	EG	38	M	30	4	−8	−21.1
LiCl	EG	24.1	M	16.4	R	−7.7	−32.0
CsF	EG	33.6	M	26	4	−7.6	−22.6
KI	EG	54	M	46.5	R	−7.5	−13.9
NaBr	EG	35.1	M	27.6	R	−7.5	−21.4
KBr	EG	43.9	M	36.7	R	−7.2	−16.4
NaI	EG	44.8	M	38	4	−6.8	−15.2
RbCl	EG	42.3	M	36	4	−6.3	−14.9
RbBr	EG	48.4	M	43	4	−5.4	−11.2
LiI	EG	38.1	M	33.2	R	−4.9	−12.9
CsI	EG	66.8	M	62	4	−4.8	−7.2
CsCl	EG	48.8	M	45	4	−3.8	−7.8
CsBr	EG	54.8	M	52	4	−2.8	−5.1
LiCl	PC	24.1	M	7	4	−17.1	−71.0
CsI	PC	66.8	M	50	4	−16.8	−25.1
CsCl	PC	48.8	M	33	4	−15.8	−32.4
NaCl	PC	29.6	M	14	4	−15.6	−52.7
RbCl	PC	42.3	M	27	4	−15.3	−36.2
KCl	PC	38	M	23	4	−15	−39.5
LiBr	PC	30.3	M	15.3	R	−15	−49.5
RbI	PC	58.9	M	44	4	−14.9	−25.3
KI	PC	54	M	39.5	R	−14.5	−26.9
LiI	PC	38.1	M	24	4	−14.1	−37.0
NaI	PC	44.8	M	31	4	−13.8	−30.8
CsBr	PC	54.8	M	43	4	−11.8	−21.5
$NaClO_4$	PC	52.9	S	41.5	R	−11.4	−21.6
RbBr	PC	48.4	M	37	4	−11.4	−23.6
NaBr	PC	35.1	M	24	4	−11.1	−31.6
KBr	PC	43.9	M	33	4	−10.9	−24.8
$CsClO_4$	PC	69.8	C	61	4	−8.8	−12.7
$RbClO_4$	PC	63.8	C	55	4	−8.8	−13.7
$LiClO_4$	PC	43.8	C	37.1	R	−6.7	−15.3
$KClO_4$	PC	55.0	C	51	4	−4.0	−7.2
KI	EC	54	M	47	4	−7	−13.0
LiI	EC	38.1	M	35	4	−3.1	−8.1
$NaClO_4$	EC	52.9	S	52	4	−0.9	−1.7
$LiClO_4$	EC	43.8	C	43	4	−0.8	−1.9
NaI	EC	44.8	M	44	4	−0.8	−1.8
$KClO_4$	EC	55.0	C	55	4	0.0	0.0
LiCl	DMSO	24.1	M	4.5	R	−19.6	−81.3
KCl	DMSO	38	M	20	4	−18	−47.4
RbF	DMSO	32.6	C	15	4	−17.6	−54.1
LiF	DMSO	11.6	M	−6	4	−17.6	−151.7
LiBr	DMSO	30.3	M	13	4	−17.3	−57.1

Volumes and error in $\mathrm{cm^3\,mol^{-1}}$.
[a]M: from molten salts[S1]; S: from soluble salts[S2]; C: calculated from density of the crystal[S3]; [b] "R" indicates a value recommended by the reviewer[S4,S5]. [c] from Ref. S6; [d] Ref. S7; [e] Ref. S8; [f] Ref. S9.

electrolyte	solvent	$\overline{V}_{\mathrm{i\ intr}}$	mode[a]	$\overline{V}_{\mathrm{i}}^{\ominus}$	minimum error[b]	$\overline{V}_{\mathrm{i\ el}}^{\ominus}$	N(% electrostr)
NaCl	DMSO	29.6	M	12.5	R	−17.1	−57.8
CsCl	DMSO	48.8	M	32	4	−16.8	−34.4
RbCl	DMSO	42.3	M	26	4	−16.3	−38.5
NaBr	DMSO	35.1	M	19.5	R	−15.6	−44.4
KBr	DMSO	43.9	M	28.4	R	−15.5	−35.3
NaF	DMSO	16.4	M	1	4	−15.4	−93.9
RbBr	DMSO	48.4	M	33	R	−15.4	−31.8
CsBr	DMSO	54.8	M	39.8	R	−15	−27.4
KF	DMSO	23.7	M	9	4	−14.7	−62.0
CsF	DMSO	33.6	M	21	4	−12.6	−37.5
CsI	DMSO	66.8	M	54.2	R	−12.6	−18.9
KI	DMSO	54	M	42.2	R	−11.8	−21.9
LiI	DMSO	38.1	M	27	4	−11.1	−29.1
NaI	DMSO	44.8	M	33.7	R	−11.1	−24.8
RbI	DMSO	58.9	M	48.2	R	−10.7	−18.2
$NaClO_4$	DMSO	52.9	S	47	4	−5.9	−11.2
$CsClO_4$	DMSO	69.8	C	67	4	−2.8	−4.1
$LiClO_4$	DMSO	43.8	C	41	4	−2.8	−6.4
$RbClO_4$	DMSO	63.8	C	61	4	−2.8	−4.3
$KClO_4$	DMSO	55.0	C	55	4	0.0	0.0
LiCl	ACE	24.1	M	−70	4	−94.1	−390.5
$LiClO_4$	ACE	43.8	C	−42	4	−85.8	−195.8
LiI	ACE	38.1	M	−31	4	−69.1	−181.4
LiBr	ACE	30.3	M	−37	4	−67.3	−222.1
NaCl	ACE	29.6	M	−26	4	−55.6	−187.8
$NaClO_4$	ACE	52.9	S	2	4	−50.9	−96.2
NaI	ACE	44.8	M	13	4	−31.8	−71.0
NaBr	ACE	35.1	M	7	4	−28.1	−80.1
RbCl	MeCN	42.3	M	−3	4	−45.3	−107.1
CsCl	MeCN	48.8	M	4	4	−44.8	−91.8
RbBr	MeCN	48.4	M	4	4	−44.4	−91.7
KCl	MeCN	38	M	−6	4	−44	−115.8
CsBr	MeCN	54.8	M	11	4	−43.8	−79.9
NaCl	MeCN	29.6	M	−14	4	−43.6	−147.3
KBr	MeCN	43.9	M	1	4	−42.9	−97.7
NaBr	MeCN	35.1	M	−7	4	−42.1	−119.9
LiCl	MeCN	24.1	M	−17	4	−41.1	−170.5
RbI	MeCN	58.9	M	18.3	R	−40.6	−68.9
NaI	MeCN	44.8	M	4.3	R	−40.5	−90.4
CsI	MeCN	66.8	M	26.4	R	−40.4	−60.5
LiBr	MeCN	30.3	M	−10	4	−40.3	−133.0
KSCN	MeCN	57.7	S	18.7	R	−39	−67.6
KI	MeCN	54	M	15.7	R	−38.3	−70.9
LiSCN	MeCN	45.5	C[e]	9	4	−36.5	−80.2
$NaClO_4$	MeCN	52.9	S	17	4	−35.9	−67.9
$RbClO_4$	MeCN	63.8	C	28	4	−35.8	−56.1
$CsClO_4$	MeCN	69.8	C	35	4	−34.8	−49.9
NaSCN	MeCN	46.6	C[f]	12	4	−34.6	−74.2
LiI	MeCN	38.1	M	5	4	−33.1	−86.9
$KClO_4$	MeCN	55.0	C	25	4	−30.0	−54.5
$LiClO_4$	MeCN	43.8	C	15.3	R	−28.5	−65.1
CsI	NMF	66.8	M	55	4	−11.8	−17.7
LiBr	NMF	30.3	M	18.7	R	−11.6	−38.3
CsBr	NMF	54.8	M	44	4	−10.8	−19.7
LiSCN	NMF	45.5	C[e]	36	4	−9.5	−20.8

Volumes and error in $\mathrm{cm^3\,mol^{-1}}$.
[a]M: from molten salts[S1]; S: from soluble salts[S2]; C: calculated from density of the crystal[S3]; [b] "R" indicates a value recommended by the reviewer[S4,S5]. [c] from Ref. S6; [d] Ref. S7; [e] Ref. S8; [f] Ref. S9.

electrolyte	solvent	$\overline{V}_{\mathrm{i\ intr}}$	mode[a]	$\overline{V}_{\mathrm{i}}^{\ominus}$	minimum error[b]	$\overline{V}_{\mathrm{i\ el}}^{\ominus}$	N(% electrostr)
KBr	NMF	43.9	M	35	4	−8.9	−20.3
CsCl	NMF	48.8	M	40	4	−8.8	−18.0
KI	NMF	54	M	45.5	R	−8.5	−15.7
NaBr	NMF	35.1	M	27.1	R	−8	−22.8
NaI	NMF	44.8	M	37.3	R	−7.5	−16.7
LiI	NMF	38.1	M	31	4	−7.1	−18.6
KCl	NMF	38	M	31	4	−7	−18.4
NaCl	NMF	29.6	M	22.7	R	−6.9	−23.3
LiCl	NMF	24.1	M	17.3	R	−6.8	−28.2
KSCN	NMF	57.7	S	51	4	−6.7	−11.6
$NaClO_4$	NMF	52.9	S	47	4	−5.9	−11.2
$CsClO_4$	NMF	69.8	C	64	4	−5.8	−8.4
$LiClO_4$	NMF	43.8	C	40	4	−3.8	−8.7
NaSCN	NMF	46.6	C[f]	43	4	−3.6	−7.7
$KClO_4$	NMF	55.0	C	55	4	0.0	0.0
LiBr	DMF	30.3	M	0.2	R	−30.1	−99.3
RbBr	DMF	48.4	M	19	4	−29.4	−60.7
RbCl	DMF	42.3	M	13	4	−29.3	−69.3
KBr	DMF	43.9	M	14.9	R	−29	−66.1
KCl	DMF	38	M	9	4	−29	−76.3
CsBr	DMF	54.8	M	26	4	−28.8	−52.6
CsCl	DMF	48.8	M	20	4	−28.8	−59.0
NaCl	DMF	29.6	M	1	4	−28.6	−96.6
LiCl	DMF	24.1	M	−3.8	R	−27.9	−115.8
NaBr	DMF	35.1	M	7.3	R	−27.8	−79.2
CsI	DMF	66.8	M	40.7	R	−26.1	−39.1
RbI	DMF	58.9	M	34.6	R	−24.3	−41.3
KI	DMF	54	M	31.3	R	−22.7	−42.0
NaI	DMF	44.8	M	22.2	R	−22.6	−50.4
LiI	DMF	38.1	M	16	4	−22.1	−58.0
$NaClO_4$	DMF	52.9	S	33	4	−19.9	−37.6
$RbClO_4$	DMF	63.8	C	45	4	−18.8	−29.4
$CsClO_4$	DMF	69.8	C	52	4	−17.8	−25.5
$LiClO_4$	DMF	43.8	C	27	4	−16.8	−38.4
$KClO_4$	DMF	55.0	C	41	4	−14.0	−25.4

Volumes and error in $cm^3\,mol^{-1}$.
[a]M: from molten salts[S1]; S: from soluble salts[S2]; C: calculated from density of the crystal[S3]; [b] "R" indicates a value recommended by the reviewer[S4,S5]. [c] from Ref. S6; [d] Ref. S7; [e] Ref. S8; [f] Ref. S9.

References

S1 T. G. Pedersen, C. Dethlefsen and A. Hvidt, *Carlsberg Res. Commun.*, 1984, **49**, 445–455.

S2 Y. Marcus, *J. Solution Chem.*, 2010, **39**, 1031–1038.

S3 CRC Press/Taylor and Francis, Boca Raton, FL, *CRC Handbook of Chemistry and Physics*, 90th edn, 2010.

S4 F. J. Millero, *Chem. Rev.*, 1971, **71**, 147–176.

S5 Y. Marcus and G. Hefter, *Chem. Rev.*, 2004, **104**, 3405–3452.

S6 C. Saunderson and R. B. Ferguson, *Acta Crystallogr.*, 1961, **14**, 321–321.

S7 R. Yode, in *e-EROS Encyclopedia of Reagents for Organic Synthesis*, John Wiley & Sons, Ltd, 2015, ch. Cesium Acetate, pp. 1–11.

S8 D. A. Lee, *Inorg. Chem.*, 1964, **3**, 289–290.

S9 VWR, *Sodium thiocyanate Density*, 2014, `https://ca.vwr.com/store/catalog/product.jsp?catalog_number=CAAA33388-A3`.

Standard molar volumes of electrolytes

protic solvents

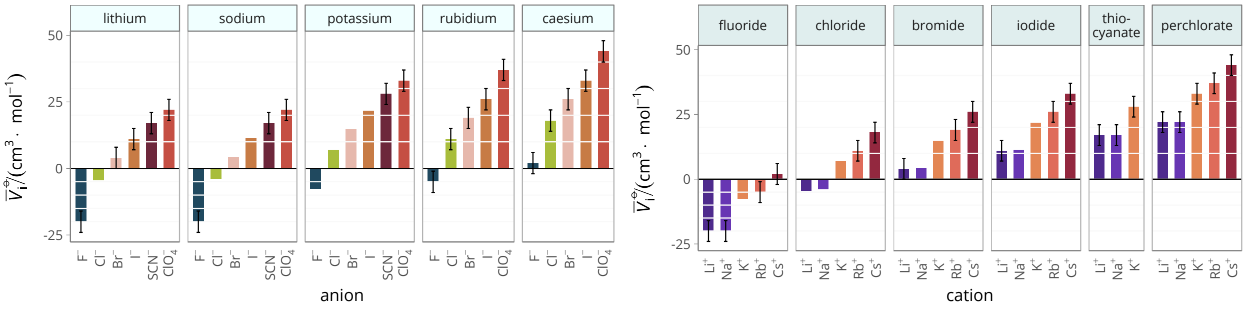

Fig. S1 Standard molar volume of alkali metal salts in methanol grouped by cation (left) and by anion (right).

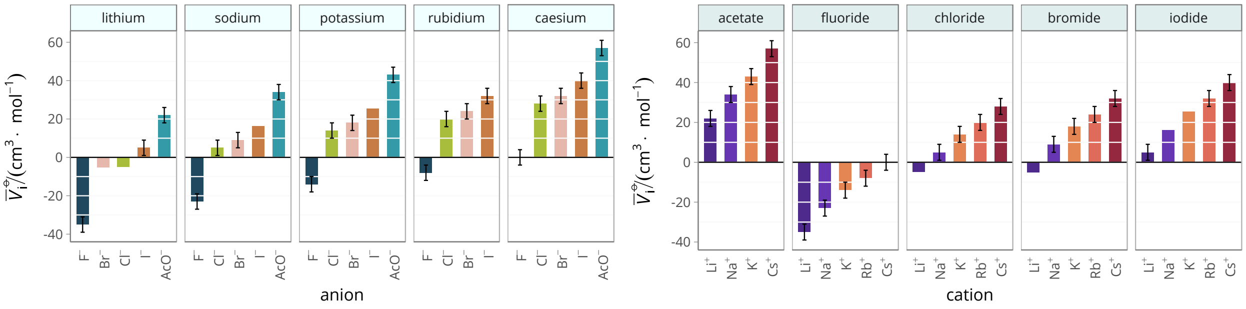

Fig. S2 Standard molar volume of alkali metal salts in ethanol grouped by cation (left) and by anion (right).

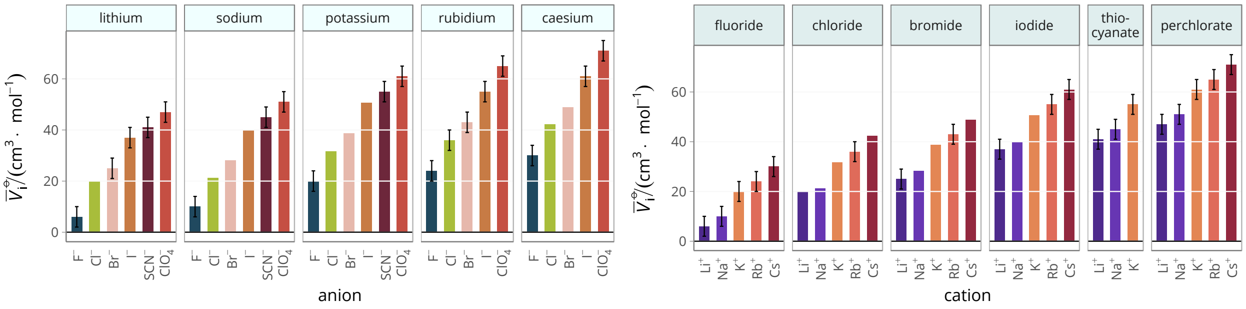

Fig. S3 Standard molar volume of alkali metal salts in formamide grouped by cation (left) and by anion (right).

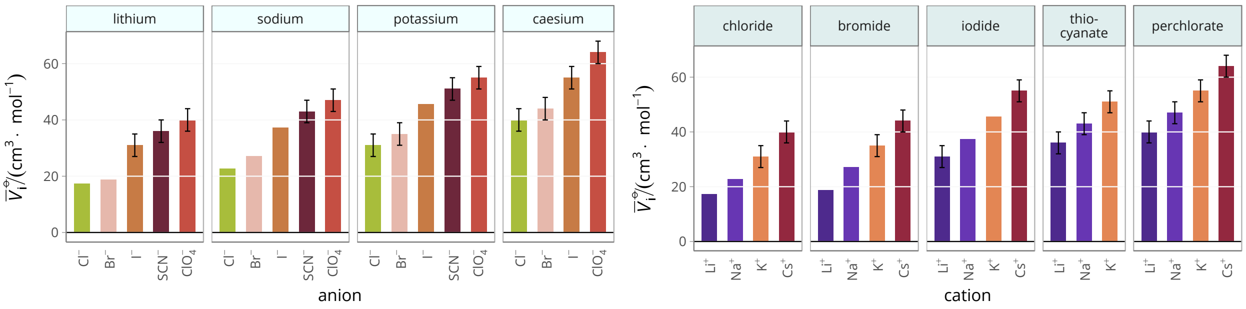

Fig. S4 Standard molar volume of alkali metal salts in *N*-Methylformamide grouped by cation (left) and by anion (right).

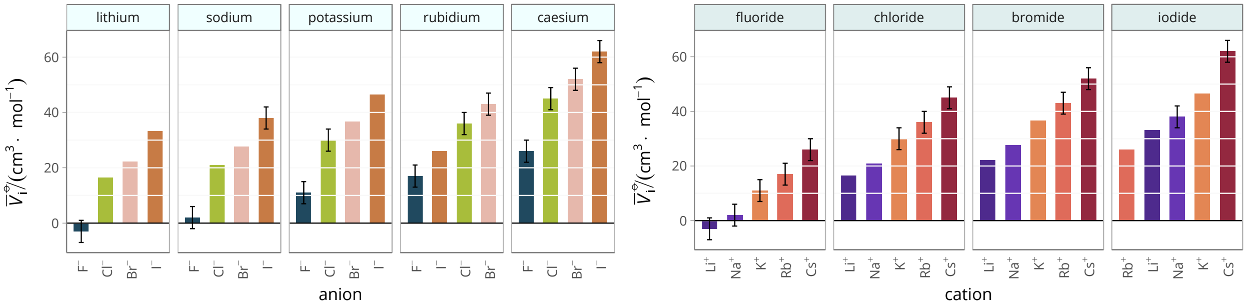

Fig. S5 Standard molar volume of alkali metal salts in ethylene glycol grouped by cation (left) and by anion (right).

aprotic solvents

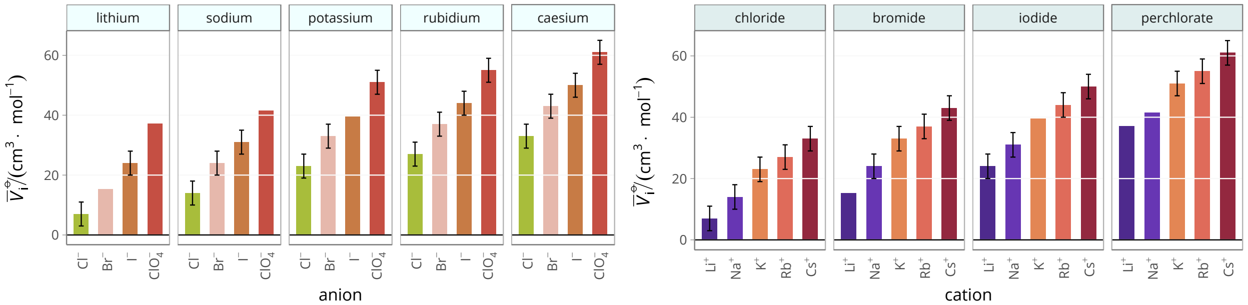

Fig. S6 Standard molar volume of alkali metal salts in propylene carbonate grouped by cation (left) and by anion (right).

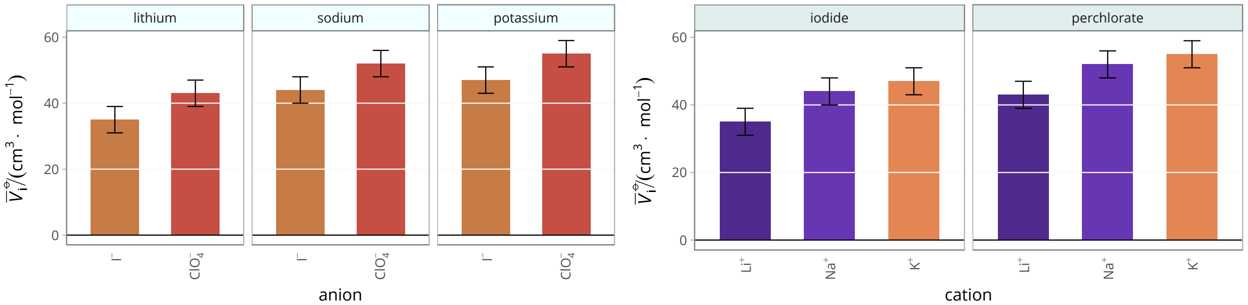

Fig. S7 Standard molar volume of alkali metal salts in ethylene carbonate grouped by cation (left) and by anion (right).

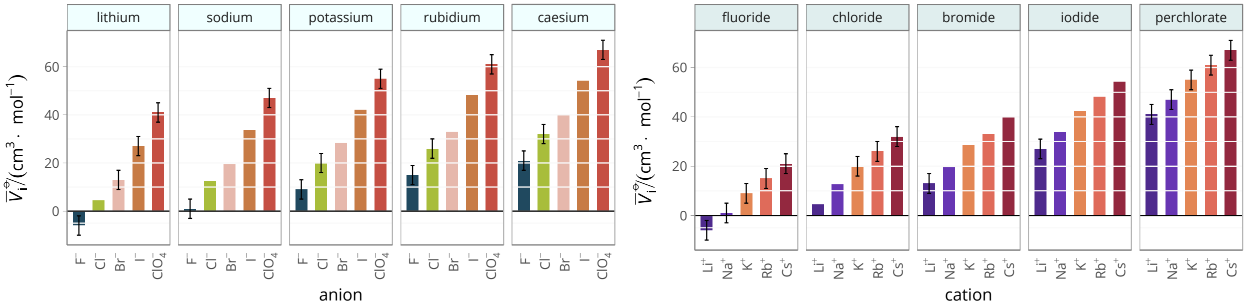

Fig. S8 Standard molar volume of alkali metal salts in dimethyl sulfoxide grouped by cation (left) and by anion (right).

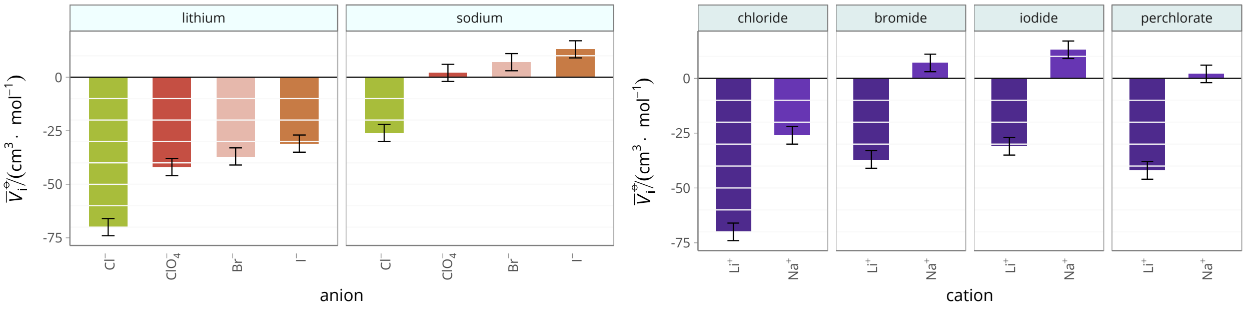

Fig. S9 Standard molar volume of alkali metal salts in acetone grouped by cation (left) and by anion (right).

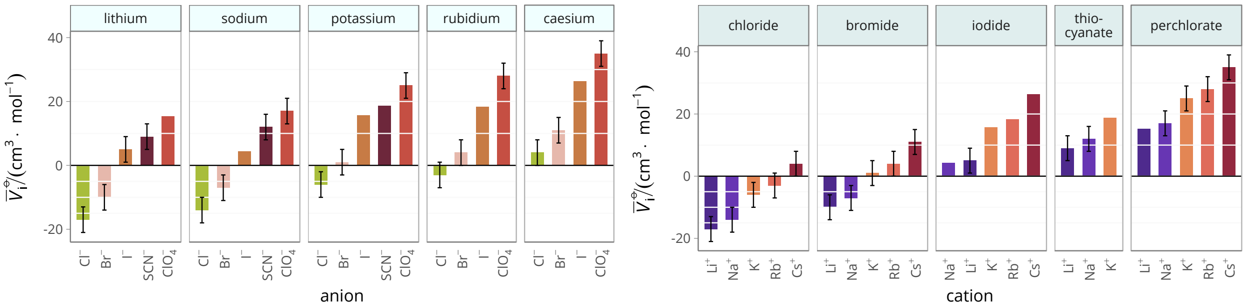

Fig. S10 Standard molar volume of alkali metal salts in acetonitrile grouped by cation (left) and by anion (right).

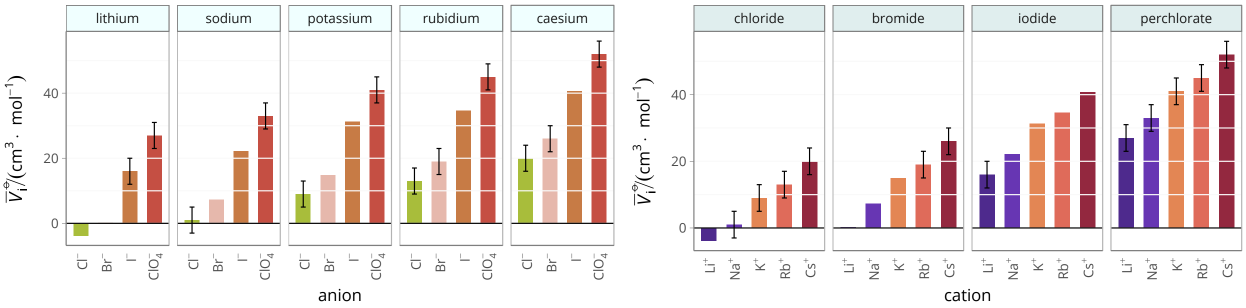

Fig. S11 Standard molar volume of alkali metal salts in *N*,*N*-Dimethylformamide grouped by cation (left) and by anion (right).

electrostrictive volume of electrolytes

protic solvents

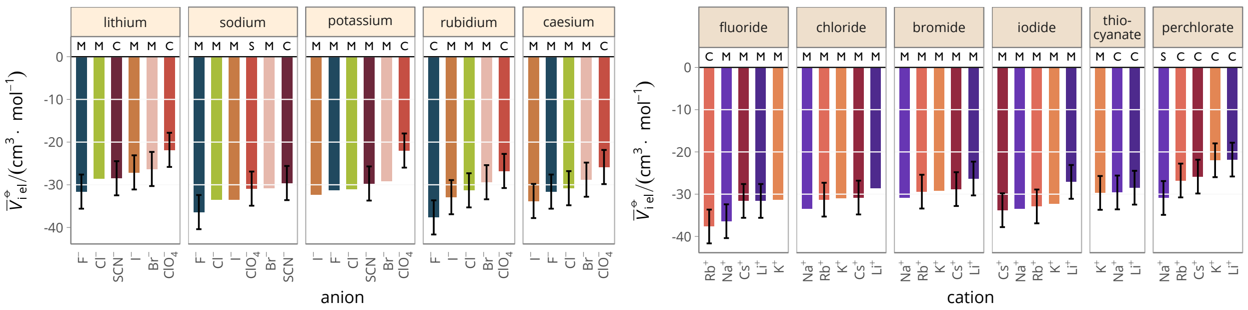

Fig. S12 Standard molar electrostrictive volume of alkali metal salts in methanol grouped by cation (left) and by anion (right).

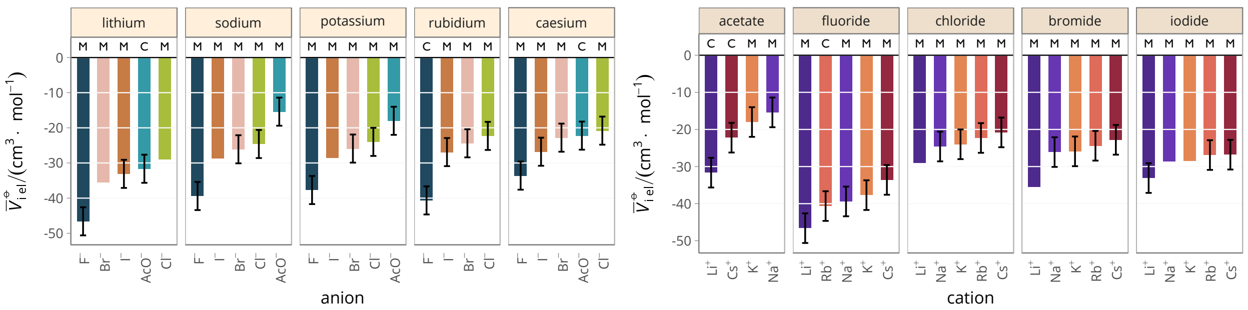

Fig. S13 Standard molar electrostrictive volume of alkali metal salts in ethanol grouped by cation (left) and by anion (right).

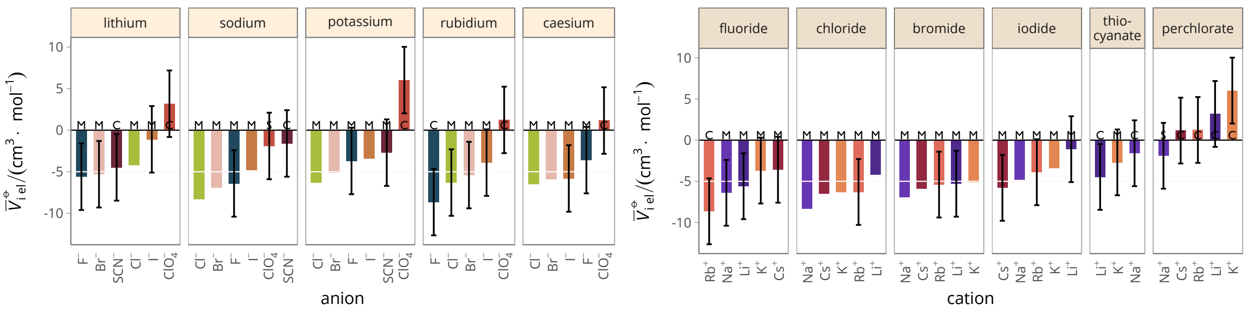

Fig. S14 Standard molar electrostrictive volume of alkali metal salts in formamide grouped by cation (left) and by anion (right).

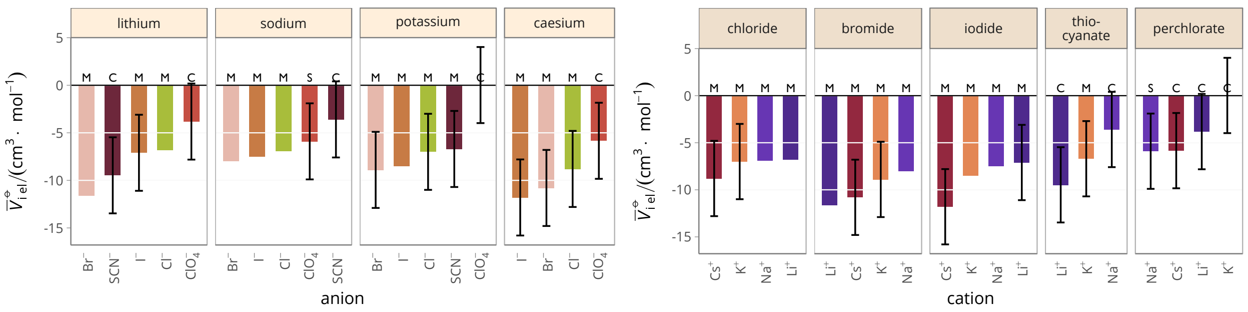

Fig. S15 Standard molar electrostrictive volume of alkali metal salts in *N*-Methylformamide grouped by cation (left) and by anion (right).

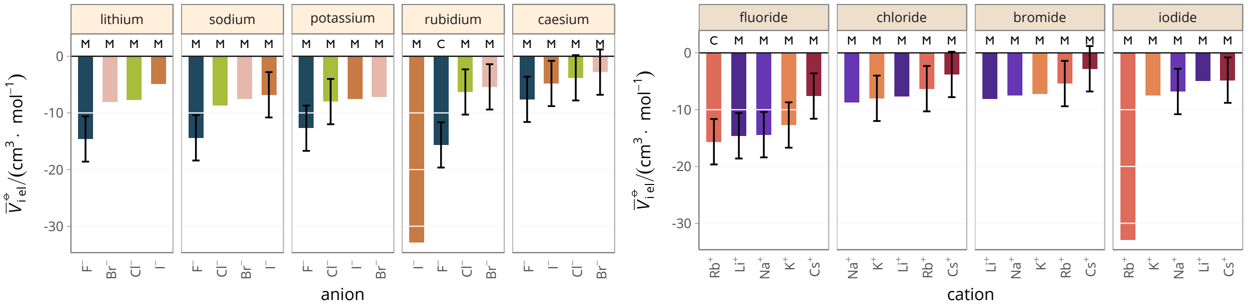

Fig. S16 Standard molar electrostrictive volume of alkali metal salts in ethylene glycol grouped by cation (left) and by anion (right).

aprotic solvents

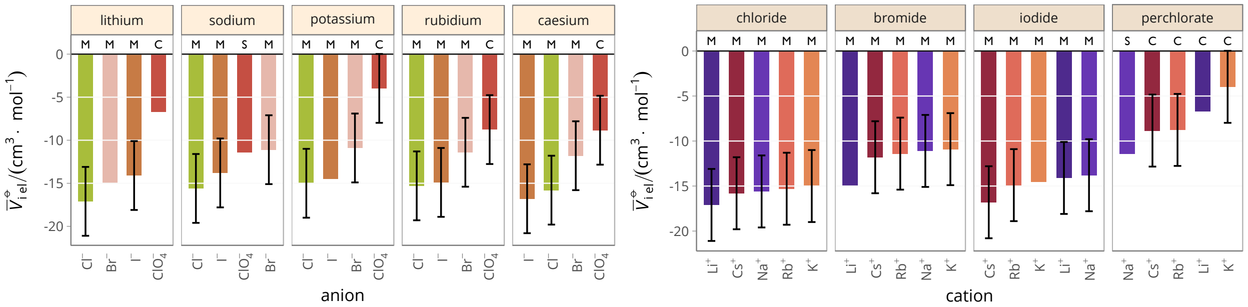

Fig. S17 Standard molar electrostrictive volume of alkali metal salts in propylene carbonate grouped by cation (left) and by anion (right).

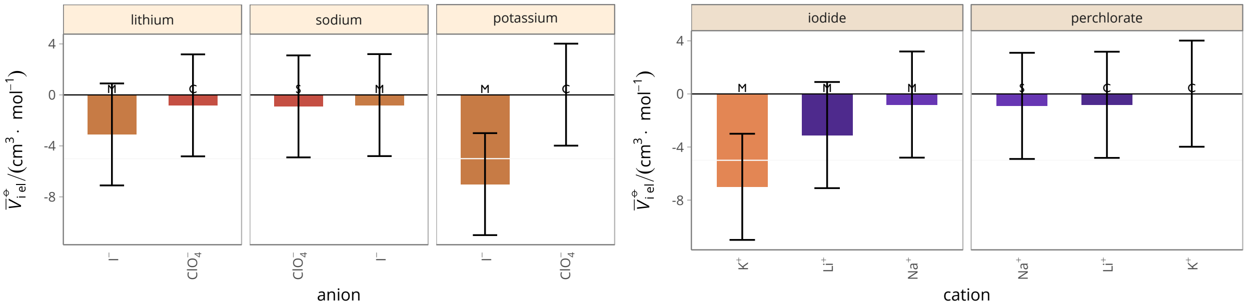

Fig. S18 Standard molar electrostrictive volume of alkali metal salts in ethylene carbonate grouped by cation (left) and by anion (right).

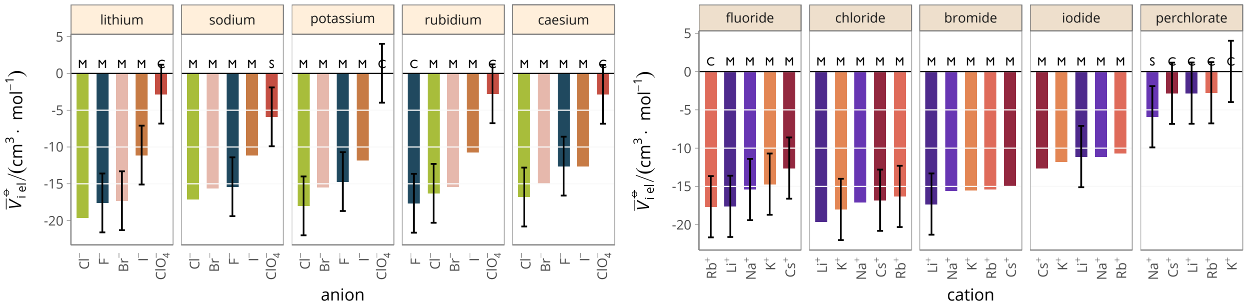

Fig. S19 Standard molar electrostrictive volume of alkali metal salts in dimethyl sulfoxide grouped by cation (left) and by anion (right).

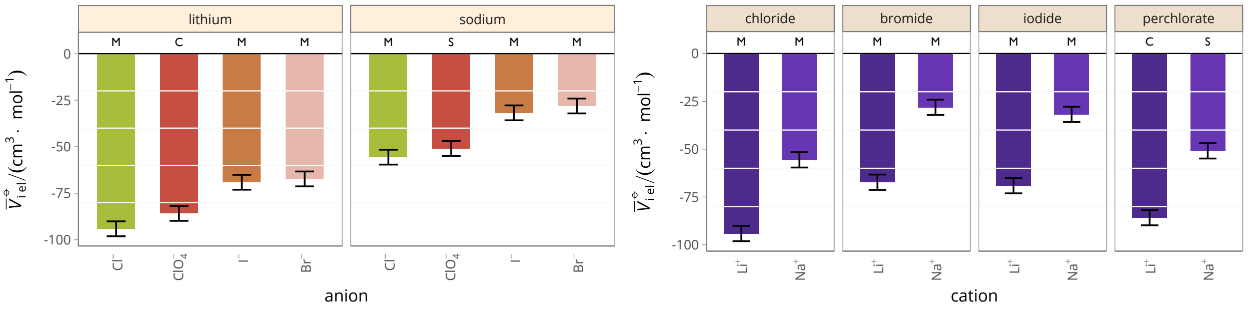

Fig. S20 Standard molar electrostrictive volume of alkali metal salts in acetone grouped by cation (left) and by anion (right).

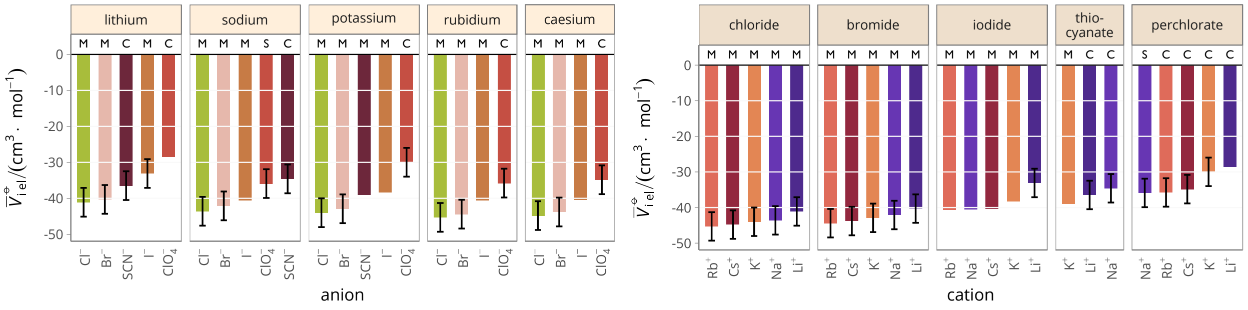

Fig. S21 Standard molar electrostrictive volume of alkali metal salts in acetonitrile grouped by cation (left) and by anion (right).

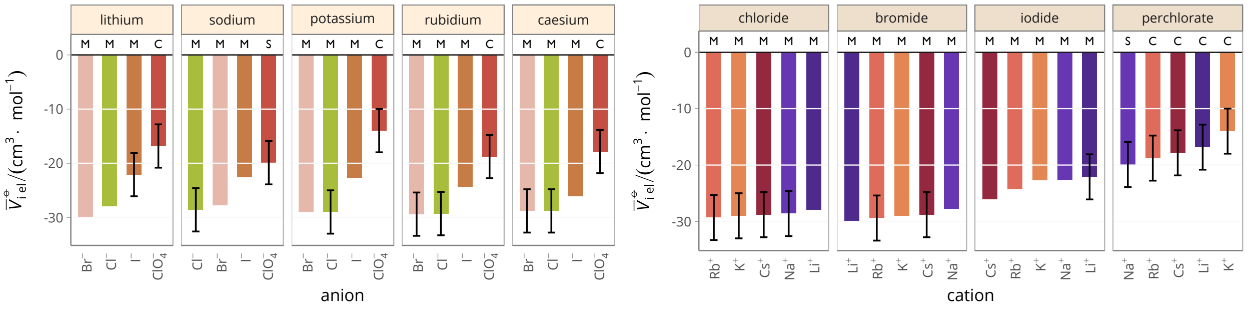

Fig. S22 Standard molar electrostrictive volume of alkali metal salts in *N*,*N*-Dimethylformamide grouped by cation (left) and by anion (right).

normalised electrostrictive volume of electrolytes

protic solvents

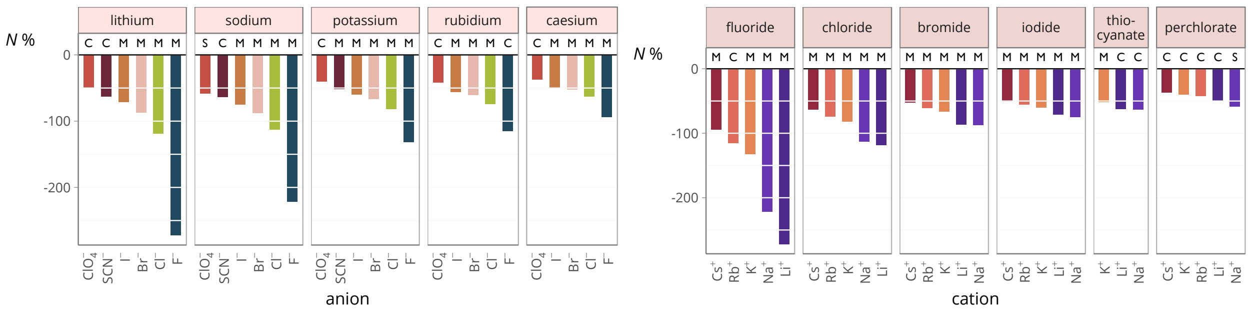

Fig. S23 Normalised standard molar electrostrictive volume of alkali metal salts in methanol grouped by cation (left) and by anion (right).

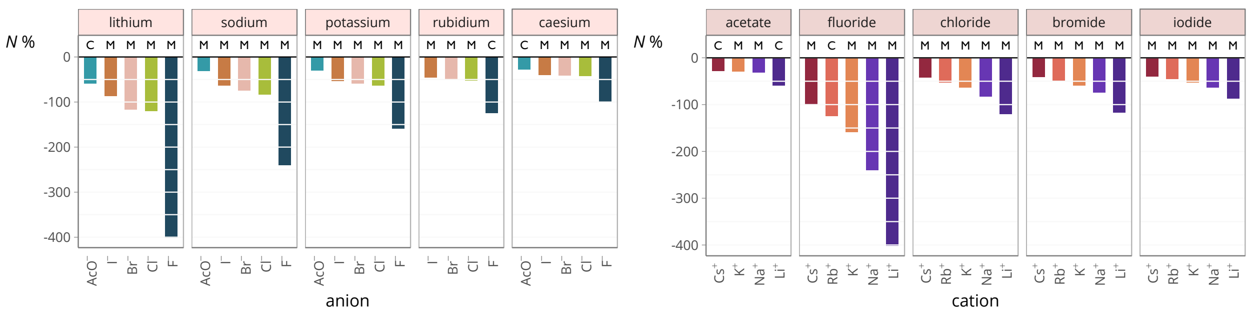

Fig. S24 Normalised standard molar electrostrictive volume of alkali metal salts in ethanol grouped by cation (left) and by anion (right).

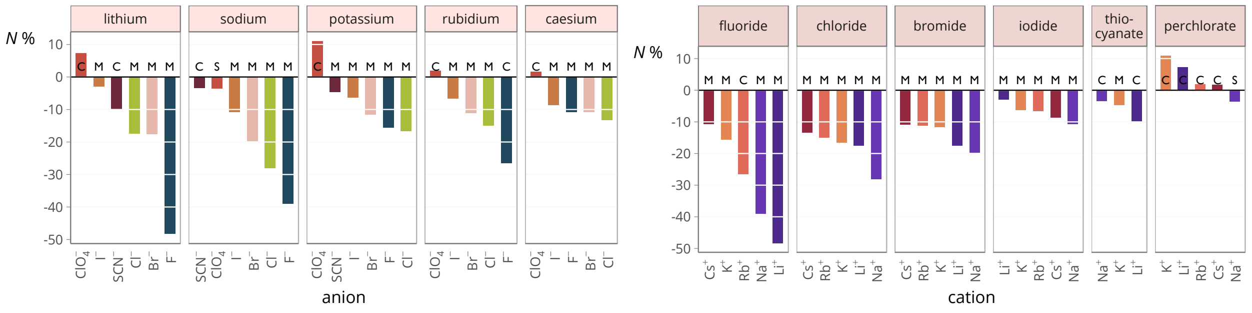

Fig. S25 Normalised standard molar electrostrictive volume of alkali metal salts in formamide grouped by cation (left) and by anion (right).

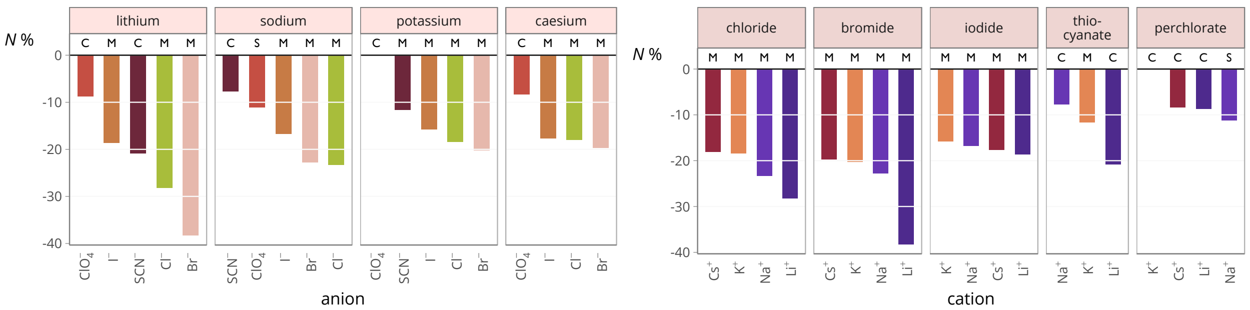

Fig. S26 Normalised standard molar electrostrictive volume of alkali metal salts in *N*-Methylformamide grouped by cation (left) and by anion (right).

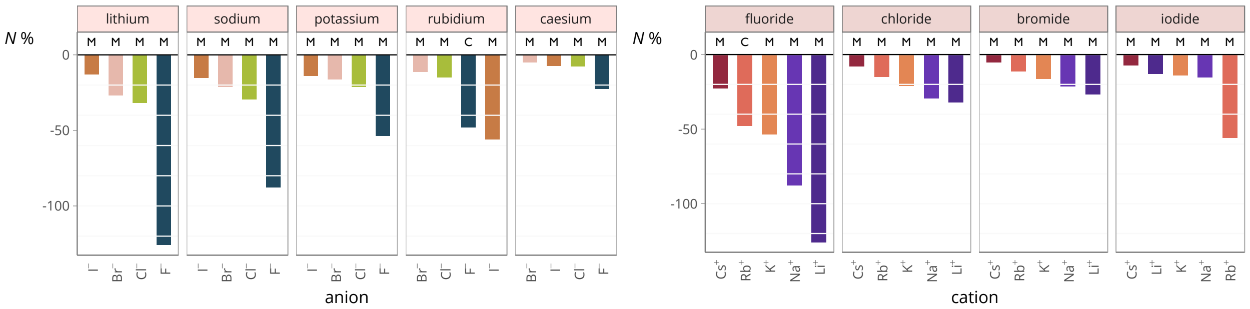

Fig. S27 Normalised standard molar electrostrictive volume of alkali metal salts in ethylene glycol grouped by cation (left) and by anion (right).

---

aprotic solvents

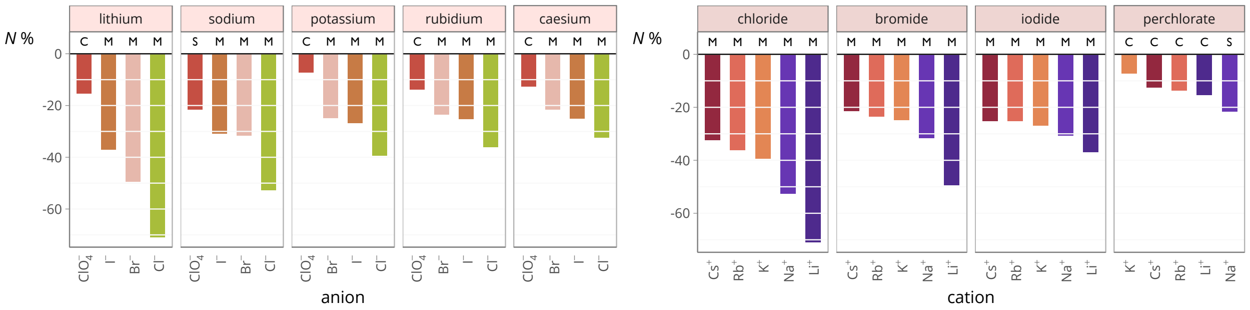

Fig. S28 Normalised standard molar electrostrictive volume of alkali metal salts in propylene carbonate grouped by cation (left) and by anion (right).

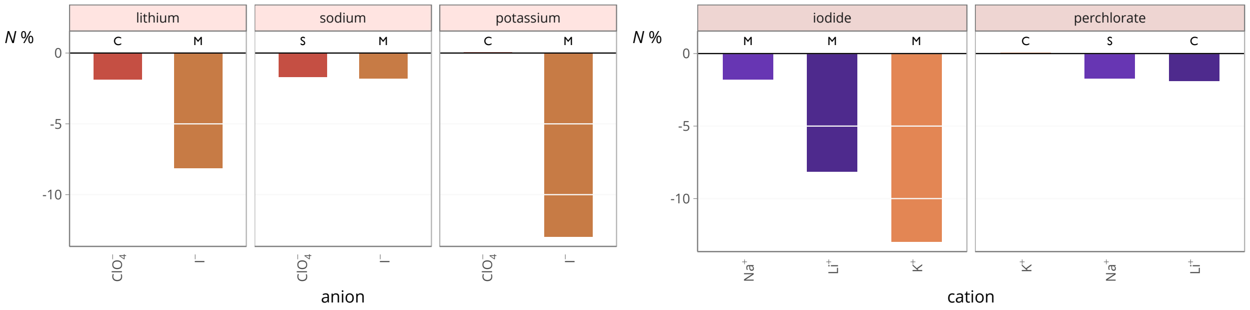

Fig. S29 Normalised standard molar electrostrictive volume of alkali metal salts in ethylene carbonate grouped by cation (left) and by anion (right).

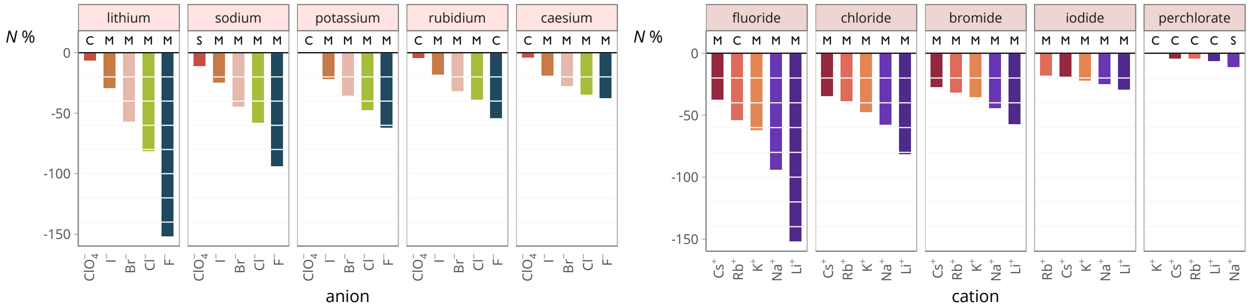

Fig. S30 Normalised standard molar electrostrictive volume of alkali metal salts in dimethyl sulfoxide grouped by cation (left) and by anion (right).

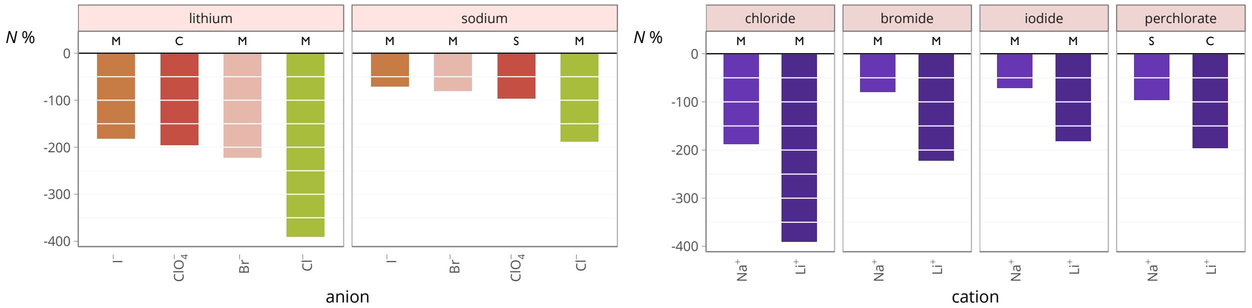

Fig. S31 Normalised standard molar electrostrictive volume of alkali metal salts in acetone grouped by cation (left) and by anion (right).

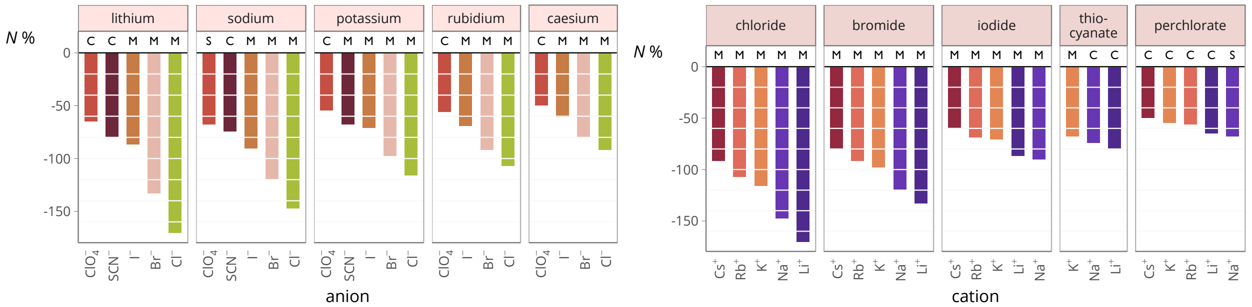

Fig. S32 Normalised standard molar electrostrictive volume of alkali metal salts in acetonitrile grouped by cation (left) and by anion (right).

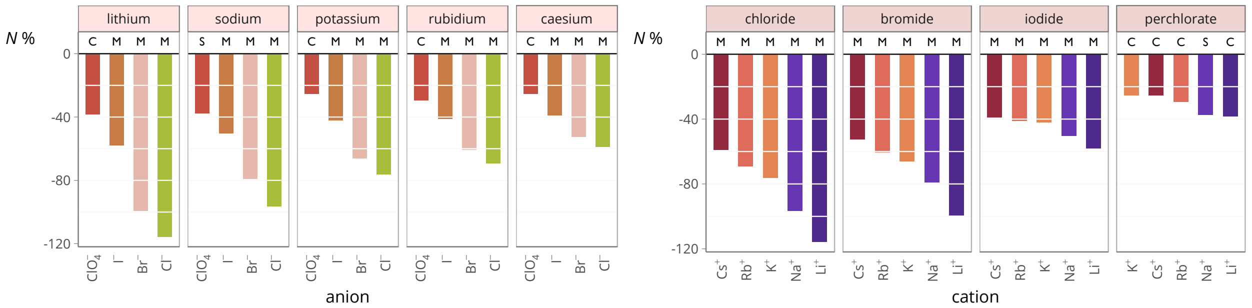

Fig. S33 Normalised standard molar electrostrictive volume of alkali metal salts in *N*,*N*-Dimethylformamide grouped by cation (left) and by anion (right).
